# Head‐to‐head comparison of relevant cell sources of small extracellular vesicles for cardiac repair: Superiority of embryonic stem cells

**DOI:** 10.1002/jev2.12445

**Published:** 2024-05-06

**Authors:** Hernán González‐King, Patricia G. Rodrigues, Tamsin Albery, Benyapa Tangruksa, Ramya Gurrapu, Andreia M. Silva, Gentian Musa, Dominika Kardasz, Kai Liu, Bengt Kull, Karin Åvall, Katarina Rydén‐Markinhuhta, Tania Incitti, Nitin Sharma, Cecilia Graneli, Hadi Valadi, Kasparas Petkevicius, Miguel Carracedo, Sandra Tejedor, Alena Ivanova, Sepideh Heydarkhan‐Hagvall, Phillipe Menasché, Jane Synnergren, Niek Dekker, Qing‐Dong Wang, Karin Jennbacken

**Affiliations:** ^1^ Research and Early Development, Cardiovascular, Renal and Metabolism, BioPharmaceuticals R&D, AstraZeneca Mölndal Sweden; ^2^ Systems Biology Research Center, School of Bioscience University of Skövde Skövde Sweden; ^3^ Department of Rheumatology and Inflammation Research, Institute of Medicine, Sahlgrenska Academy University of Gothenburg Gothenburg Sweden; ^4^ AstraZeneca India Private Limited, Neville Tower 11th Floor, Ramanujan IT SEZ Rajv Gandhi Salai (OMR), Taramani Chennai Tamil Nadu India; ^5^ Discovery Sciences, Oligo Assay Development BioPharmaceuticals R&D, AstraZeneca Mölndal Sweden; ^6^ Anjarium Biosciences AG Schlieren Switzerland; ^7^ Pharmaceutical Sciences, Advanced Drug Delivery, BioPharmaceuticals R&D, AstraZeneca Mölndal Sweden; ^8^ Chief Medical Office, Global Patient Safety, AstraZeneca Mölndal Sweden; ^9^ Department of Cardiovascular Surgery, Hôpital Européen Georges Pompidou Université de Paris, PARCC, INSERM Paris France; ^10^ Department of Molecular and Clinical Medicine, Institute of Medicine, Sahlgrenska Academy University of Gothenburg Gothenburg Sweden

**Keywords:** angiogenesis, fibrosis, immunomodulation, myocardial ischaemia‐reperfusion injury, regeneration, small extracellular vesicles

## Abstract

Small extracellular vesicles (sEV) derived from various cell sources have been demonstrated to enhance cardiac function in preclinical models of myocardial infarction (MI). The aim of this study was to compare different sources of sEV for cardiac repair and determine the most effective one, which nowadays remains limited. We comprehensively assessed the efficacy of sEV obtained from human primary bone marrow mesenchymal stromal cells (BM‐MSC), human immortalized MSC (hTERT‐MSC), human embryonic stem cells (ESC), ESC‐derived cardiac progenitor cells (CPC), human ESC‐derived cardiomyocytes (CM), and human primary ventricular cardiac fibroblasts (VCF), in in vitro models of cardiac repair. ESC‐derived sEV (ESC‐sEV) exhibited the best pro‐angiogenic and anti‐fibrotic effects in vitro. Then, we evaluated the functionality of the sEV with the most promising performances in vitro, in a murine model of MI‐reperfusion injury (IRI) and analysed their RNA and protein compositions. In vivo, ESC‐sEV provided the most favourable outcome after MI by reducing adverse cardiac remodelling through down‐regulating fibrosis and increasing angiogenesis. Furthermore, transcriptomic, and proteomic characterizations of sEV derived from hTERT‐MSC, ESC, and CPC revealed factors in ESC‐sEV that potentially drove the observed functions. In conclusion, ESC‐sEV holds great promise as a cell‐free treatment for promoting cardiac repair following MI.

## 1 INTRODUCTION

Heart failure is a major cause of morbidity and mortality worldwide, affecting more than 60 million patients within the last decade (Yan et al., 2023). Although several drugs and mechanical devices that can improve cardiac function have been developed (Mancini & Burkhoff, 2005), none of them have been able to promote the restoration of non‐functional heart tissue into new functional cardiac tissue, which is the key cause underlying heart failure. In response to injury, the endogenous repair process of the heart involves a limited division of preexisting cardiomyocytes (Bearzi et al., 2007; Senyo et al., 2013). Evidence suggests that enhanced angiogenesis immediately after myocardial infarction (MI) can rescue myocytes from death and prevent adverse cardiac remodelling (Gogiraju et al., 2019; Johnson et al., 2019). Moreover, in the mammalian heart, after MI, millions of cardiomyocytes are lost and replaced by fibrotic scar tissue (Kuppe et al., 2022). Therefore, stimulation of therapeutic angiogenesis, regulation of fibrotic scar formation, and the replacement of injured tissue with new cardiomyocytes are considered key processes in promoting cardiac repair and preventing the development of heart failure after MI (Bubb et al., 2019; Z. Li et al., 2019). Growing evidence suggests that stem cell therapy may be a promising approach for the formation of new functional cardiac cells and improvement of angiogenesis (Poch et al., 2022).

Small extracellular vesicles (sEV) have been identified as major mediators of stem cell‐induced effects in the heart after injury (Clark et al., 2014; Phinney & Pittenger, 2017). sEV are membrane vesicles with less than 200 nm in diameter that are secreted by cells and carry membrane and cytosolic proteins, metabolites and nucleic acids, including mRNAs, microRNAs (miRNAs), and long non‐coding RNAs, that can be transferred into recipient cells and trigger downstream functions (Boulanger et al., 2017; Tejedor et al., 2023). Previous studies have shown that extracellular vesicles (EVs) isolated from various cell types, such as mesenchymal stromal cells (MSC), embryonic stem cells (ESC) or induced pluripotent stem cells (iPSC), cardiospheres, cardiac progenitor cells (CPC), epicardium derived cells (EDC) and cardiomyocytes (CM), improve cardiac function after MI through cardiac repair (Chen et al., 2020; Del Campo et al., 2022; Khan et al., 2015; C. Li, Ni et al., 2021). However, the specific EVs components responsible for the observed effects have not been clearly identified and a comprehensive comparison of the various effects that EVs from different cell sources may have in cardiac repair is still missing.

In this study, we aimed to compare relevant sources of sEV and determine the most effective source from a cardiac repair perspective. To achieve this, we assessed the efficacy of sEV obtained from primary human bone marrow MSC (BM‐MSC), human MSC immortalized by ectopic expression of human telomerase reverse transcriptase (hTERT‐MSC), human ESC, ESC‐derived CPC and CM, in key processes of cardiac repair. We further investigated the composition and functionality of the three most promising sEV candidates in a murine model of myocardial ischaemia‐reperfusion injury (IRI). Our findings showed that ESC‐sEV were the most effective in reducing adverse cardiac remodelling after MI by decreasing fibrosis and increasing angiogenesis in the ischaemic heart. In addition, we performed a large‐scale transcriptomic and proteomic characterization of the top sEV candidates by applying RNA‐seq and proteomics analysis. These data revealed associations between the sEV content and the observed effects in vitro and in vivo.

## 2 METHODS

The human cell lines employed in this study for sEV isolation encompassed VCF and BM‐MSC from LONZA, hTERT‐MSC from ATCC, and ESC (SA121 cell line) from TaKaRa. The generation of CPC and CM was realized via differentiation of the ESC cell line. Briefly, sEVs originating from VCF, BM‐MSC, and hTERT‐MSC were isolated from their respective maintenance media, which were supplemented with EV‐free FBS and previously incubated with the cells for a duration of 48 h. ESC‐sEVs were extracted from their maintenance media following a 24‐h incubation period. The isolation of CPC‐sEVs was achieved using conditioned media from days 5 to 8 of ESC differentiation into CM, while CM‐sEVs were isolated from the conditioned media of ESC‐derived CM between days 15 to 25 of ESC differentiation into CM. The sEVs sourced from the diverse cell origins and for the different functional assays performed were purified using a serial ultracentrifugation protocol. However, to quantitate the yield of sEVs per millilitre of conditioned media from the various cell sources, the isolation was carried out using size exclusion chromatography columns, specifically the IZON qEV10 column. The total protein content of the isolated sEVs was quantified using the QubitTM protein quantification kit from ThermoFisher Scientific. The same amount of sEV total protein from distinct cell sources was employed for all experimental analyses. The sEV functional roles were assessed across in vitro models representing angiogenesis, fibrosis, cardioprotection, proliferation, and modulation of macrophage responses. Based on the outcomes derived from these in vitro experiments, hTERT‐MSC, ESC, and CPC were identified as the prime candidates. These top candidates underwent further evaluation within an in vivo model involving myocardial ischaemia‐reperfusion injury in mice. Additionally, the RNA and protein compositions of these candidates were characterized using transcriptomics and proteomics methodologies.

Comprehensive details concerning aspects such as cell culture procedures, sEV isolation techniques, transmission electron microscopy, nanoparticle analysis, western blotting, RNA extraction, in vitro and in vivo functional assays, as well as the acquisition and analysis of proteomic and RNA‐seq data, histological evaluations, and statistical analyses performed throughout this study are elucidated within the supplementary material. sEV work was conducted having into account MISEV guidelines (Welsh et al., 2024).

## 3 RESULTS

### 3.1 Molecular characterization of sEV cell sources and sEV isolates

**FIGURE 1:**
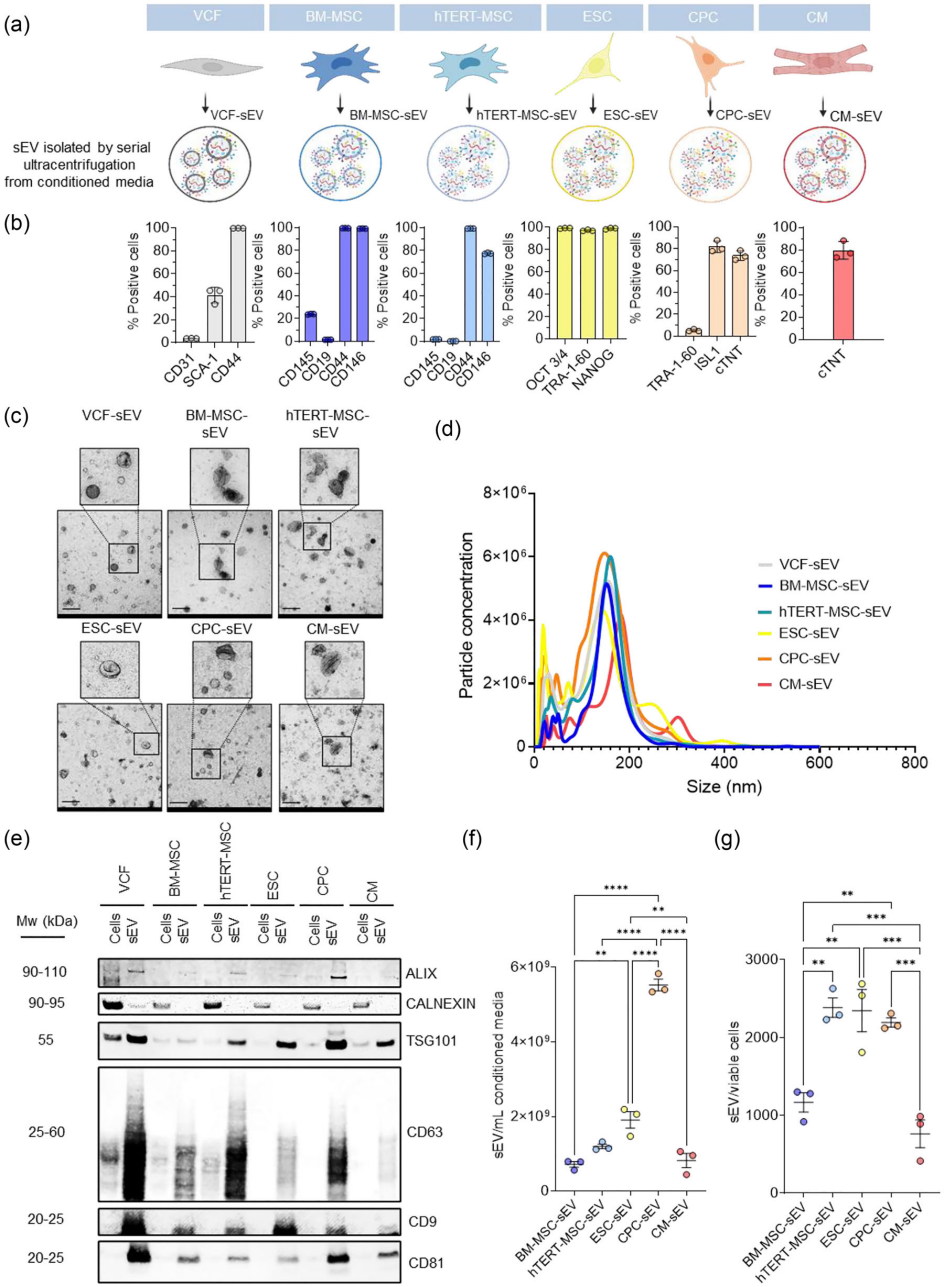
Characterizations of vesicles isolated from various cell sources. (a) Schematic representation of the different cell sources used for sEV preparation. (b) Bar graphs showing the percentage of cells expressing each indicated cell type‐specific marker measured by flow cytometry. (c) Electron microscopy images of isolated sEV. Enlarged images highlight detailed vesicles morphology. Scale bar: 200 nm. (d) sEV size distribution analysis with the NanoSight LM14c. (e) Western blot analysis for ALIX, CALNEXIN, TSG101, CD63, CD9 and CD81 in protein extracts from indicated sEV and their parental cells. Five µg of total cell or sEV protein was loaded in the Western blot for every sample. (f) sEV yield per mL of conditioned media from indicated cell sources. (g) sEV yield per viable cell from the indicated cell sources. *p < 0.05; **p < 0.01; ***p < 0.001; ****p < 0.0001. One‐way ANOVA followed by Tukey post‐hoc test were applied. Dots in the bar graphs represent biological replicates. *N* = 3. MW (Molecular weight).

We first performed quality control on the cell sources used for sEV isolation by evaluating their expression of protein markers previously described to be enriched in each cell type by flow cytometry (Figure 1a). hTERT‐MSC was included to overcome the scalability limitation of primary BM‐MSC. Ninety‐nine percent of BM‐MSC expressed CD44 and CD146 and 0% of them expressed CD19. Ninety‐nine percent and 77% of hTERT‐MSC expressed CD44 and CD146, respectively, but none expressed both CD145 and CD19. Undifferentiated ESC expressed pluripotency markers OCT 3/4, TRA‐1‐60, and NANOG whereas on day 7 of CPC differentiation, 81% of the cells expressed the CPC marker ISL‐1, 73% expressed the CM marker cTNT, and 5% expressed the undifferentiated ESC marker TRA‐1‐60. Partial expression of SCA‐1 together with high expression of CD44 and lack of the expression of CD31 have been reported as a characteristic phenotypic profile of VCF (Stellato et al., 2019). Forty‐two percent and 99% of VCF expressed SCA‐1 and CD44, respectively, whereas none expressed CD31.

We then isolated sEV from the conditioned media from different cell sources by ultracentrifugation and characterized these sEV by transmission electron microscopy (TEM), nanoparticle tracking analysis (NTA), and western blotting. TEM analysis revealed that all sEV prepared from different cell sources had a typical cup‐shape morphology characteristic of TEM‐processed sEV samples and there were no visible differences among them (Figure 1c). Particle analysis showed a predominance of vesicles within 80 and 200 nm (Figure 1d). Western blot analysis of sEV and lysates of their parental cells demonstrated enrichment of proteins typically associated with sEV, such as ALIX, TSG101, CD63, CD9, and CD81, in vesicle isolates from the different cell sources. In contrast, the endoplasmic marker CALNEXIN was enriched in the cell lysates and was barely detectable in vesicle isolates (Figure 1e). Moreover, within this study, we standardized the treatments by normalizing them with the total small extracellular vesicle (sEV) protein across the various types of sEV utilized. Given that the protein content in sEV isolates may vary based on the isolation method employed and sEV type, we performed a correlation analysis between the particle count and micrograms of total sEV protein for the specific sEV types under investigation (Figure S1). Notably, no significant differences were observed in the particle count per microgram of total sEV protein across the diverse sEV types.

To quantify the number of sEV obtained per mL of conditioned media produced by the different cell sources and to avoid the variability introduced by the serial ultracentrifugation protocol, we isolated sEV by size exclusion chromatography using IZON columns. We used the same volume of conditioned media from three different cultures of the different parental cell types under their optimal growing conditions and quantified the number of particles with NTA that CPC and ESC conditioned media yielded the highest amounts of sEV (Figure 1f). We also calculated the number of sEV generated per viable cell after 48 h of culture (Figure 1g), finding again hTERT‐MSC, ESC and CPC as producing the higher number of sEV.

### 3.2 ESC‐sEV shows pro‐angiogenic and anti‐fibrotic properties, but not cardioprotective effects, in cardiac in vitro models

**FIGURE 2:**
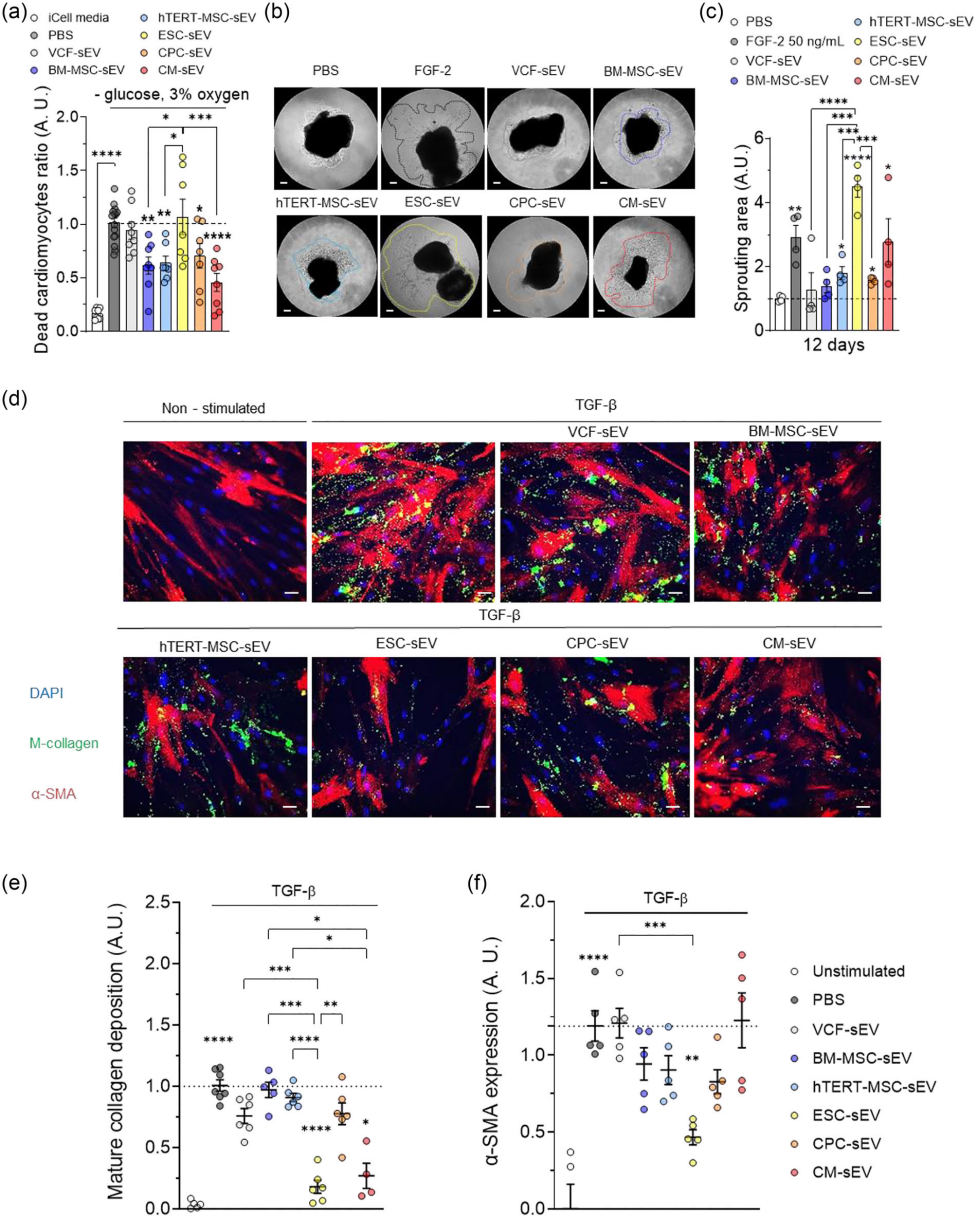
Assesing the effects of sEV in in vitro models of cardiomyocyte ischemia and cardiac fibrosis, and in an ex vivo model of angiogenesis. (a) Dead cardiomyocytes ratio in an in vitro model of cardioprotection measured by the % of nuclei that incorporate propidium iodide after treatment with PBS (dashed line), the indicated types of sEV, or iCell media. (b) Bright field microscopy images of mouse aortic rings 12 days after being embedded in Matrigel and treated with PBS, 25 ng/ml FGF‐2 or the indicated types of sEV. In the representative images, it is possible to observe the sprouts grown from the rings into the Matrigel, delineated within dotted lines. Scale bar: 200 µm. (c) Quantification of the area covered by the sprouts grown from the aortic rings into the Matrigel in response to the different treatments on day 12. Dashed line represents the sprouting area of the PBS‐treated rings. (d) Representative fluorescence microscopy pictures of VCF 48 h after being stimulated with the pro‐fibrotic cocktail (TGF‐β, dextrane sulfate and L‐ascorbic acid) in a low serum culture medium (0.5% of FBS) in the absence or presence of sEV from indicated origins. VCF nuclei are stained with Hoechst (blue) while their mature collagen (M‐collagen, green) and alpha‐smooth muscle actine (α‐SMA, red) are immunostained to monitor the pro‐fibrotic phenotype (dashed line). Non‐stimulated: VCF cultured in standard conditions without the addition of pro‐fibrotic cocktail as the negative control. Scale bar: 20 µm. (e and f) Graphs showing the quantification of M‐collagen (e) or α‐SMA (f) in the VCF under different conditions. *p < 0.05; **p < 0.01; ***p < 0.001; ****p < 0.0001. One‐way ANOVA followed by Dunnett or Tukey's post‐hoc test. Dots in the bar graphs represent all the technical replicates (n). *N* = 4 in each experiment.

We first evaluated the cardioprotective effects of sEV derived from different cell sources on hiPSC‐CM under hypoxic and starvation conditions. We found that sEV from hTERT‐MSC, BM‐MSC, CPC, and CM reduced cell death by nearly two‐fold whereas ESC‐sEV had no effect compared to PBS control (Figure 2a). As expected, VCF‐sEV did not affect cardiomyocyte death and non‐starving cardiomyocytes in iCell media were prevented from death.

Next, we examined the pro‐angiogenic properties of sEV using two different models of angiogenesis, that is, capillary‐like tube formation by human cardiac microvascular endothelial cells (hCMVE‐C) in Matrigel and mouse aortic rings. FGF‐2 and VEGF‐A were used as positive controls in the aortic ring and tube formation assays, respectively. hCMVE‐C generated higher, and similar, numbers of loops within the matrigel in response to all sEV types compared to the vehicle group (PBS) (Figure S2A and S2B). However, ESC‐sEV had the greatest capacity to induce sprout formation in the more physiologically relevant ex vivo model of aortic ring assay compared to sEV from VCF, BM‐MSC, hTERT‐MSC and CPC (Figure 2b,c). Furthermore, the percentage of proliferative hCMVE‐C, measured as cells expressing KI67, was also increased after treatment with sEV from BM‐MSC, hTERT‐MSC, ESC, CPC, or CM (Figure S2C and S2D). Lastly, we evaluated the anti‐fibrotic properties of sEV released from different cell sources in an in vitro model of cardiac fibrosis using VCF. Our results indicated that only the ESC‐sEV treatment reduced the expression of both mature collagen and α‐SMA whereas CM‐sEV reduced the expression of mature collagen but not α‐SMA (Figure 2d–f).

### 3.3 sEV from BM‐MSC, hTERT‐MSC, ESC, CPC and CM reduce M1 features in M1 macrophages

**FIGURE 3:**
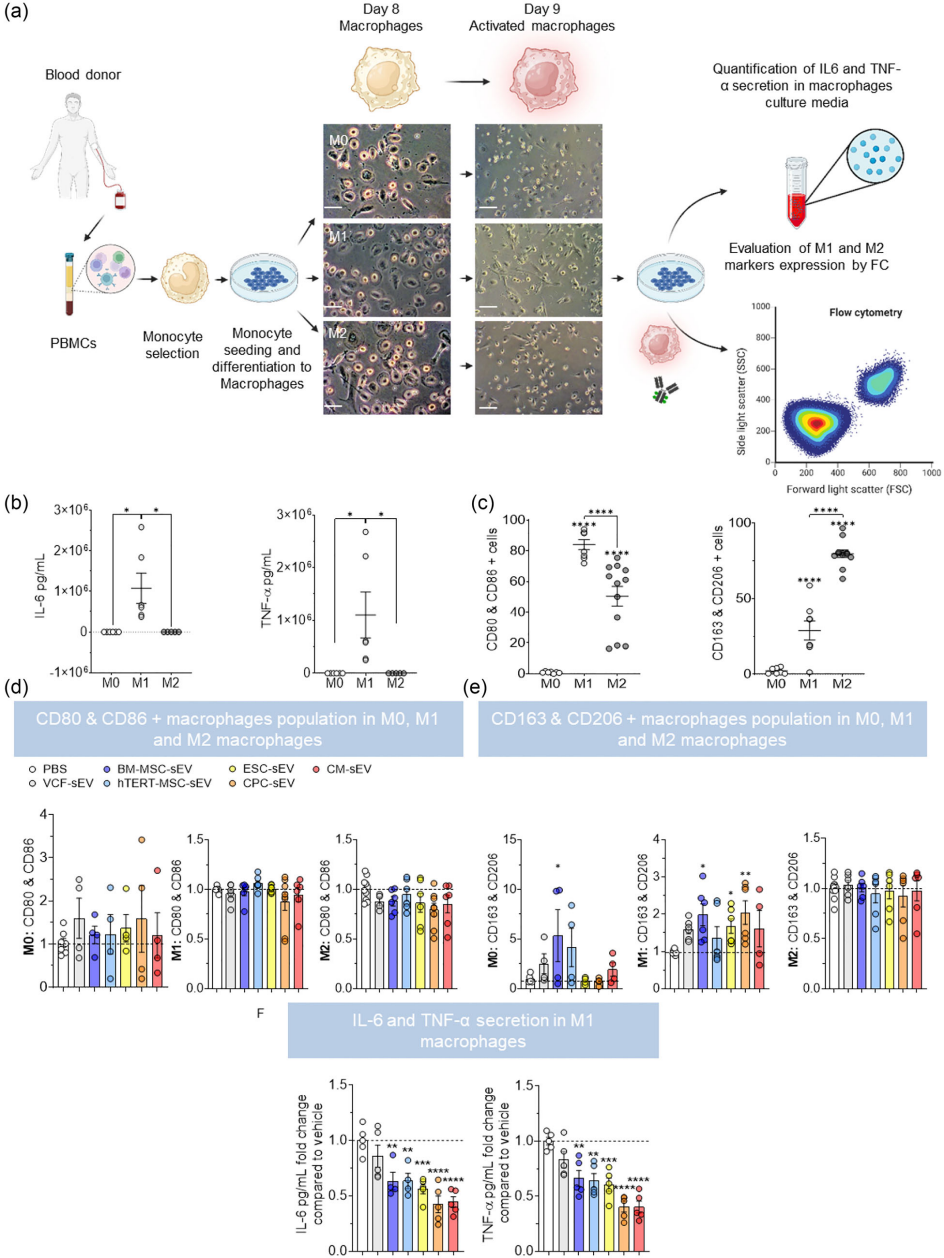
Promotion of M1 to M2 transition and reduction of pro‐inflammatory cytokine secretion by sEV. (a) Schematic representation of the experimental setup. Monocytes are isolated from PBMCs obtained from the blood of healthy donors, seeded in culture plates, differentiated into M0, M1 and M2 polarized macrophages, and treated with sEV on days 6 and 8 of the differentiation. On day 8, the media are also supplemented with IFN‐γ and LPS for M1 macrophages or IL4 and IL13 for M2 macrophages. On day 9, conditioned media from macrophages is collected to measure secreted cytokines and macrophages are harvested to evaluate their expression of M1 and M2 polarization markers by flow cytometry. Scale bar from bright field pictures from the left: 30 µm; from the right: 60 µm. (b) Graphs showing the amount of IL‐6 (left) and TNF‐α (right) secreted by M0, M1 and M2 macrophages treated with PBS. (c) Graphs showing the percentage of indicated macrophage subtypes expressing M1 markers, CD80 and CD86, or M2 markers, CD163 and CD206, measured by flow cytometry. (d) Expressions of M1 markers CD80 and CD86 in M0, M1 and M2 macrophages treated with sEV from indicated cell sources. (e) Expressions of M2 markers CD163 and CD206 in M0, M1 and M2 macrophages treated with sEV from the indicated cell sources. (f) IL‐6 and TNF‐α secretion in M1 macrophages treated with the different types of sEV. All graphs in d, e and f, represent fold change of positive cells to PBS‐treated control (dashed lines). *p < 0.05; **p < 0.01; ***p < 0.001; ****p < 0.0001. One‐way ANOVA multiple comparisons analysis followed by two‐stage‐step‐up method of Benjamini, Krieger and Yekutieli post‐hoc test were applied (Benjamini et al., 2006). Dots in the bar graphs represent all the technical replicates (n). *N* = 3.

To evaluate effects of sEV on macrophages, we differentiated human monocytes into macrophages, polarized the macrophages into M0, M1, and M2 phenotypes, and activated them with the corresponding cytokines (LPS and IFN‐γ for M1, and IL‐4 and IL‐13 for M2) at day 8 of differentiation. We treated the M0, M1 and M2 macrophages with sEV or PBS (control) before (day 6) and at the beginning of their activation (day 8). Then, we evaluated the release of the pro‐inflammatory cytokines (IL‐6 and TNF‐α) by the macrophages and measured their expression of M1/M2 markers expression at day 9 of differentiation (Figure 3a). M0 and M2 macrophages released very low levels of TNF‐α and IL‐6 whereas M1 macrophages released higher levels of IL‐6 and TNF‐α (Figure 3b). M0 macrophages had almost undetectable levels of double positive markers for M1 (CD80 and CD86) or M2 (CD163 and CD206) cells (Figure 3c). Both M1 and M2 macrophages expressed M1 and M2 markers but a higher percentage of M1 macrophages expressed M1 markers compared to M2 macrophages, whereas a higher percentage of M2 macrophages expressed M2 markers compared to M1 macrophages (Figure 3c).

The expression of M1 or M2 markers in M2 polarized macrophages was not affected by sEV treatment. However, treating with sEV from BM‐MSC increased the percentage of M0 macrophages expressing M2 markers and treating with sEV from BM‐MSCs, ESC, and CPCs increased the percentage of M1 macrophages expressing M2 markers (Figure 3d,e). Treating M1 macrophages with sEV derived from hTERT‐MSC, BM‐MSCs, ESC, CPC, and CM reduced their secretion of IL‐6 and TNF‐α (Figure 3f). However, treating M0 and M2 macrophages with sEV from different parental cell sources did not change their cytokine release (Figure S3A and S3B).

### 3.4 hTERT‐MSC, ESC and CPC‐derived sEV induce cardiomyocyte proliferation

Based on the above in vitro results and their scalability potential, we determined that hTERT‐MSC, ESC and CPC‐sEV were the most promising candidates for further evaluation in vitro and in vivo.

We wanted to investigate the capacity of the top sEV to induce cardiomyocyte cell cycle activity by measuring EdU incorporation in hiPSC‐CM. All three top sEV, hTERT‐MSC‐sEV, ESC‐sEV and CPC‐sEV, induced EdU incorporation in hiPSC‐CM (Figure S4A and S4B).

### 3.5 ESC‐sEV attenuates cardiac remodelling after MI in mice through pro‐angiogenic and anti‐fibrotic effects

**FIGURE 4:**
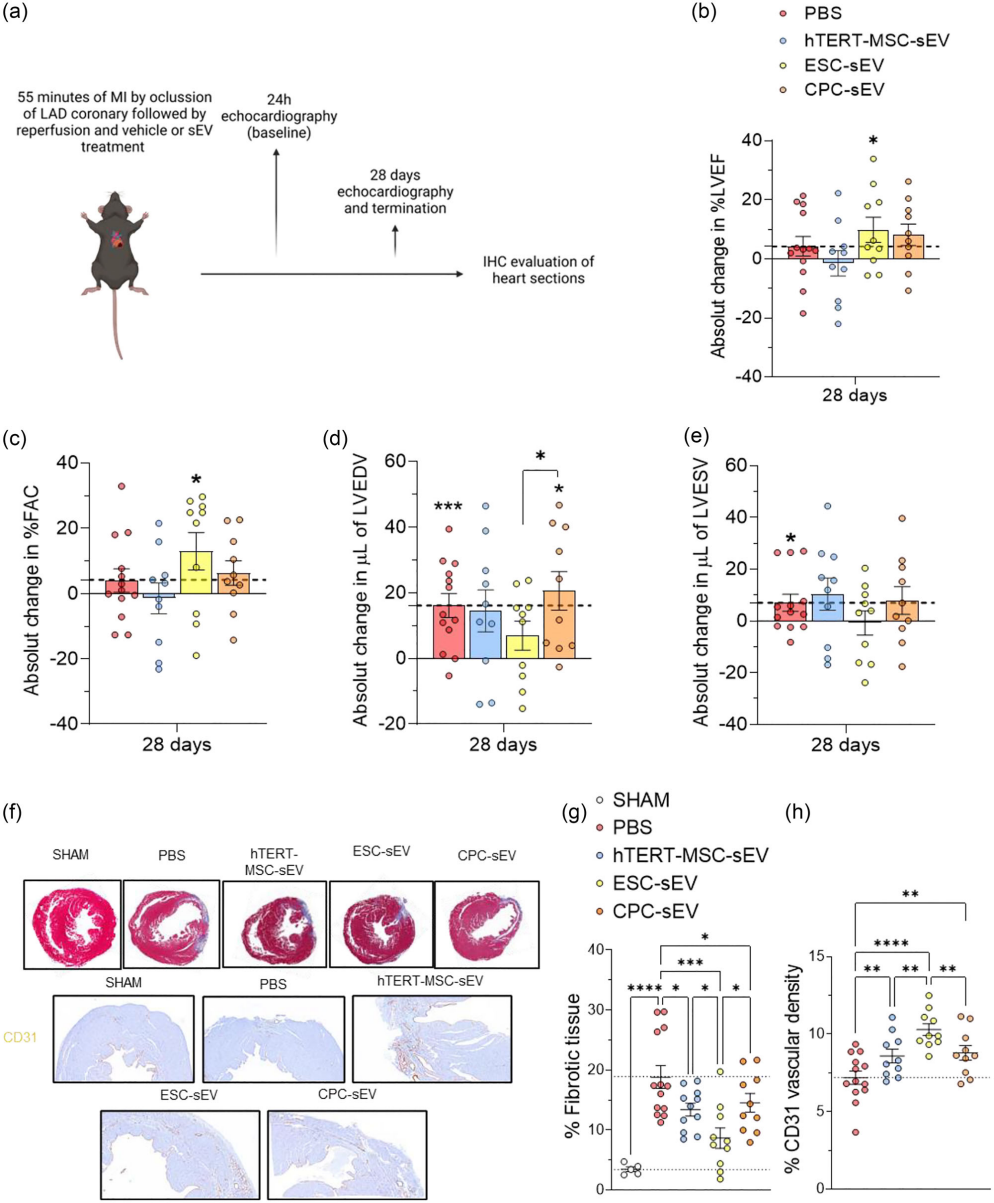
Treatment with ESC‐sEV improves cardiac function and reduces adverse cardiac remodelling following myocardial ischaemia‐reperfusion injury. (a) Schematic representation of the experimental setup for the evaluation of sEV function in an in vivo model of myocardial ischaemia and reperfusion injury. (b‐e) Echocardiography data showing the absolute change on LVEF (b, in percentage), FAC (c, in percentage), LVEDV (d, in µl) and LVESV (e, in µl) at 28 days versus 24 h (dashed lines) after MI and reperfusion without (PBS) or with treatment with hTERT‐MSC‐sEV, ESC‐sEV or CPC‐sEV. (f) Representative images showing Masson trichrome (top row) and anti‐CD31 immunostaining (lower two rows) of hearts harvested 28 days after M‐IR from the five experimental groups, sham, PBS, hTERT‐MSC‐sEV, ESC‐sEV and CPC‐sEV. (g and h) Percentages of fibrotic tissue (g, blue staining in F) and vascular density (h, brown staining in F) of heart sections. The statistical significance comparing the basal value to the endpoint value within each condition is indicated at the top of each group. *p < 0.05; **p < 0.01; ***p < 0.001; ****p < 0.0001. A two‐way ANOVA mixed effects analysis followed by two‐stage‐step‐up method of Benjamini, Krieger and Yekutieli post‐hoc test was applied. Dots in the bar graphs represent biological replicates (N). *N* = 13 in PBS group and 10 on sEV treatments groups.

We used hTERT‐MSC, ESC and CPC‐sEV as acute treatments in a murine model of myocardial ischaemia and reperfusion injury by directly injecting them into the border zone of the infarcted myocardium (Figure 4a). At 28 days after MI, echocardiography data revealed that hearts of mice treated with the vehicle (PBS) did not exhibit any improvement in left ventricle ejection fraction (LVEF) or fractional area change (FAC) and showed significantly increased left ventricle end‐diastolic volume (LVEDV) and left ventricle end‐systolic volume (LVESV) compared to the baseline (24 h after MI) (Figure 4b‐e and Figure S5). In contrast, mice treated with ESC‐sEV exhibited an increase in absolute changes in LVEF and FAC while maintaining LVEDV and LVESV at levels similar to those of the baseline 28 days following MI. Neither LVESV in hTERT‐MSC or CPC‐sEV treatment groups nor LVEDV in the hTERT‐MSC‐sEV treatment group exhibited a significant increase 28 days after MI compared to the baseline (Figure 4b‐e). Consistent with these findings, histological evaluation of the infarcted hearts revealed that all, ESC‐sEV, CPC‐sEV and hTERT‐MSC‐sEV, reduced the percentage of fibrotic tissue and increased vasculature in the MI area, with ESC‐sEV showing the strongest effects (Figure 4f‐h).

### 3.6 Multi‐OMICs and functional enrichment analysis of hTERT‐MSC, ESC and CPC‐sEV content correlates with their in vitro and in vivo performance

To characterize the cargo of sEV produced by hTERT‐MSC, ESC and CPC, we used transcriptomic and proteomic approaches. We also compared the transcriptome and proteome of the sEV with that of their parental cells. To minimize the risk of DNA contamination, DNAse was used to digest any potential contaminant DNA present in our samples during RNA extraction. No extensive large DNA was detected, and the profile of small‐sized nucleic acids was not changed after DNAse treatment (Figure S6A).

Capillary electrophoresis revealed that the RNA from sEV was enriched in fragments smaller than 1000 nt whereas the RNA from cells was characterized by the presence of the typical 28S and 18S rRNA peaks and a small fraction of small RNAs (∼100‐200 nt; Figure S6B,C). Additionally, the RNA profile of sEV secreted by different cell types exhibited distinctive features, only ESC‐sEV carried significant amounts of transcripts with sizes up to 1000 nt whereas hTERT‐MSC and CPC‐sEV contained mainly RNAs up to 200 nt (Figure S6B).

**FIGURE 5:**
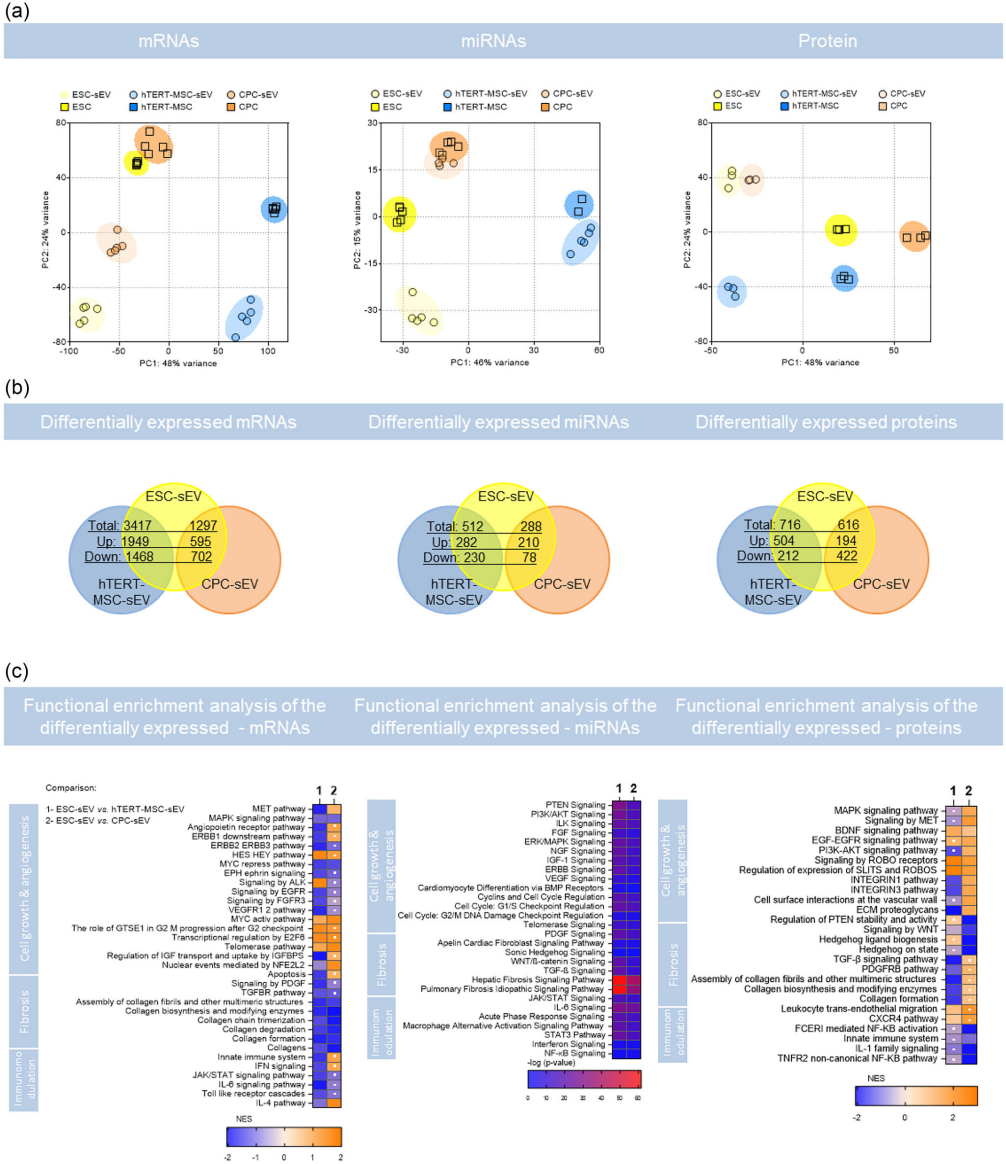
Functional enrichment analysis of differentially expressed RNAs and proteins in sEV. (a) PCA of mRNA, miRNA, and protein compositions of ESC (yellow), CPC (orange) and hTERT‐MSC (blue) and their respective sEV (light yellow, orange, and blue, respectively). (b) Venn diagram of mRNAs, miRNAs and proteins differentially expressed in ESC‐sEV versus hTERT‐MSC‐sEV or versus CPC‐sEV. The tabulation for the differentially expressed mRNAs or miRNAs is a *p*‐value < 0.05 and |log2FC| > 2.5, and for proteins is a *p*‐value < 0.05 and |log2FC| > 0.5. (c) Heatmaps normalized enrichment score (NES) results from the differentially expressed mRNAs, miRNAs, and proteins enrichment analysis of canonical pathways for comparing ESC‐sEV versus hTERT‐MSC‐sEV (indicated as 1) or versus CPC‐sEV (indicated as 2). Non‐significant NES is identified with a white square in the heatmap. Square and circles in the PCA represent biological replicates (N). *N* = 5 for mRNA and miRNA samples, *N* = 3 for protein samples.

Interestingly, principal component analysis (PCA) of the multi‐omics datasets revealed different clustering of the samples at transcriptome and proteome levels. The transcriptome similarity of ESC and CPC cells and their corresponding sEV samples dominates when compared to hTERT‐MSC samples. This was observed both at the mRNA and at the miRNA levels and represents 48% (mRNA) and 46% (miRNA) of the variance in the data respectively (Figure 5a).

On the other hand, the PCA of the proteomics data showed a higher similarity between sEV from different sources than to their respective parental cell proteome, which clustered together and separated from the sEV (Figure 5a).

Differential expression analysis showed that differentially expressed mRNAs and miRNAs molecules were mostly up‐regulated in sEV compared to their parental cells, except for miRNAs from CPC samples, where a similar number of up‐ and down‐regulated miRNAs were observed when sEV were compared to their parental cells (Figure S7A and S7B). In contrast, differentially expressed proteins were down‐regulated in sEV compared to their parental cells (Figure S7C).

The in vitro and in vivo functional evaluation of sEV from different sources showed that ESC‐sEV demonstrated the most promising pro‐angiogenic and anti‐fibrotic properties among the investigated sEV (Figures 2 and 4). To investigate the molecular differences that may underpin these differences we performed differential expression analysis comparing ESC‐sEV with hTERT‐MSC‐sEV and CPC‐sEV. Fewer differentially expressed mRNAs, miRNAs, or proteins were observed between ESC‐sEV and CPC‐sEV (1, 297; 288; 615, respectively) than between ESC‐sEV and hTERT‐MSC‐sEV (3,147; 512; 715, respectively) (Figure 5b and S8A).

Functional annotation of significantly regulated canonical pathways was performed using the fgsea package and canonical pathway gene sets downloaded from the Molecular Signatures Database (MsigDB) or using the core‐ and Comparison‐Analysis functions from Ingenuity Pathway Analysis software, respectively. The GSEA Normalized Enrichmend Score (NES), for mRNAs and proteins, or *p*‐values for miRNAs, were plotted in a heatmap (Figure 5c). The enriched pathways were manually classified into cell growth and angiogenesis, fibrosis, or immunomodulatory‐related pathways. The NES scores provide information about the directionality of the regulation, and this is not provided with the *p*‐values. VEGFR1 2 pathway was down‐regulated in ESC‐sEV differentially expressed mRNAs. However, other pathways that could potentially contribute to their pro‐angiogenic effect were up‐regulated compared to hTERT‐MSC‐sEV and CPC‐sEV, like ROBO, BDNF, MYC or telomerase, related pathways. Fibrosis‐related pathways like collagen metabolism, WNT, TGF‐β or PDGF signalling pathways, showed to be down‐regulated in ESC‐sEV. Processes related to inflammation like the Innate immune system response, or IFN‐γ, IL‐6 and IL‐1 signalling pathways, tend to be down‐regulated in ESC‐sEV, except for those linked to leukocytes extravasation or CXCR4 that appear up‐regulated in comparison to hTERT‐MSC‐sEV. Furthermore, differentially expressed miRNAs in ESC‐sEV compared to hTERT‐MSC‐sEV and CPC‐sEV were enriched for pathways linked to cell growth and angiogenesis, fibrosis or immunomodulation.

**FIGURE 6:**
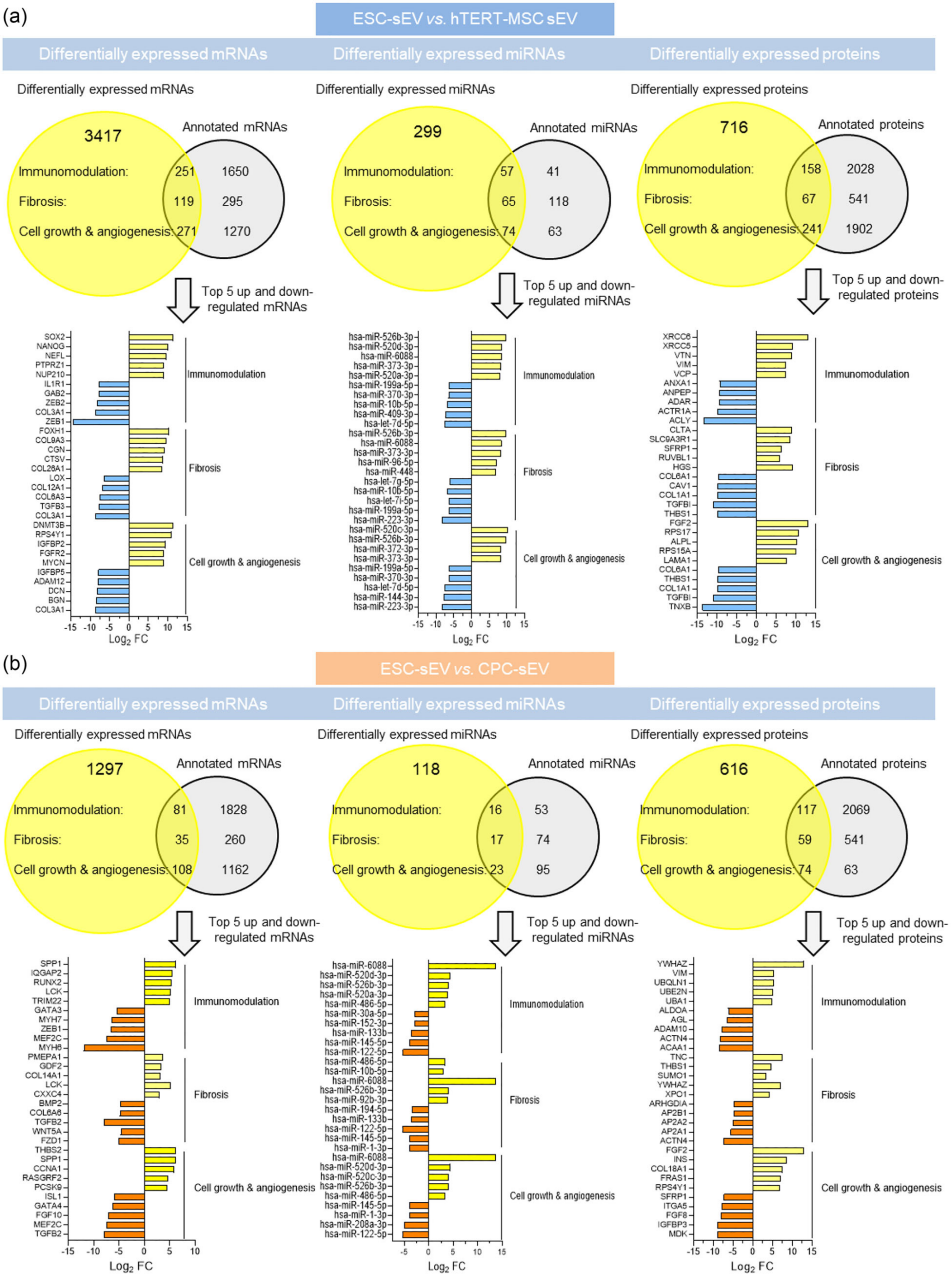
Functional classification of differentially expressed mRNAs, miRNAs, and proteins. (a and b) Numbers of differentially expressed and identified mRNAs, miRNAs, and proteins in ESC‐sEV versus hTERT‐MSC‐sEV (a) or versus CPC‐sEV (b) are shown in the Venn diagrams. The tabulation for the differentially expressed mRNAs or miRNAs is *p*‐value < 0.05 and |log2FC| > 2.5 and for proteins is *p*‐value < 0.05 and |log2FC| > 0.5. The top 5 up‐regulated and top 5 down‐regulated mRNAs, miRNAs, or proteins involved in the regulation of cell growth & angiogenesis, fibrosis, and the immune system represented with the Log_2_ FC (Log_2_ fold change) in the “x” axis for ESC‐sEV versus hTERT‐MSC‐sEV (a; yellow for ESC‐sEV and blue for hTERT‐MSC‐sEV) or versus CPC‐sEV (b; yellow for ESC‐sEV and orange for CPC‐sEV) are shown at the bottom. Annotated mRNAs/miRNAs/proteins: molecules that have been identified in the literature to regulate the immune system, fibrosis, or cell growth and angiogenesis.

Figure 6a,b shows the top five most up‐regulated and down‐regulated mRNAs, proteins and miRNAs in the ESC‐sEV versus hTERT‐MSC‐sEV and ESC‐sEV versus CPC‐sEV that participate in the regulation of the mentioned pathways of interest. To gain a more comprehensive understanding of the abundance of the most differentially expressed molecules, Figure S9 illustrates the quantity of the differentially expressed mRNAs, miRNAs, and proteins, enriched in ESC‐sEV.

## 4 DISCUSSION

Given the post‐mitotic nature of cardiomyocytes and their limited ability to proliferate, dead cardiomyocytes after MI are typically replaced by non‐contractile fibrotic tissue. To improve cardiac recovery following MI, it is crucial to minimize cardiomyocyte loss, stimulate cardiomyocyte proliferation directly or through reparative mechanisms such as angiogenesis or regulating the fibrotic scar formation (Beltrami et al., 2003; Khan et al., 2015; Senyo et al., 2013). Our findings reveal that sEV from hTERT‐MSC, BM‐MSC, CPC, and CM, but not from ESC, protect cardiomyocytes from apoptosis induced by oxygen, serum, and glucose deprivation. In contrast, ESC‐sEV demonstrates the strongest pro‐angiogenic and anti‐fibrotic effects in vitro. Furthermore, sEV from hTERT‐MSC, ESC, and CPC increase the percentage of hiPSC‐CM entering the cell cycle in vitro. However, further investigation is needed to confirm whether this effect signifies true adult cardiomyocyte proliferation, that is, cytokinesis, rather than binucleation or nuclear polyploidization in both in vitro and in vivo settings (Kadow & Martin, 2018). Macrophage phenotype regulation, especially the transition from a pro‐inflammatory (M1) to a pro‐reparative (M2) state, has been identified as a critical process affecting MI outcomes (Lima Correa et al., 2021; Yap et al., 2023). Notably, we observed that sEV derived from BM‐MSC, ESC, and CPC promoted polarization to the M2 phenotype under M1 polarizing conditions that mimic the early inflammatory stages following MI (Hoyer & Nahrendorf, 2019; King et al., 2017). Additionally, all screened sEV, except for the negative control VCF‐sEV, reduced the secretion of pro‐inflammatory cytokines IL‐6 and TNF‐α by M1 macrophages. In summary, our in vitro results demonstrate that ESC‐sEV exhibit superior reparative properties by directly regulating angiogenesis and fibrosis. In addition, ESC‐sEV shows the potential to promote cardiac repair indirectly by shifting macrophage from a pro‐inflammatory to a reparative phenotype. This is in line, with previous investigations that showed in vitro that hiPSC‐derived sEV had a greater activity in inducing tube formation with human endothelial cells and cell cycle in hiPSC‐CM compared to CPC‐sEV or CM‐sEV, but we extended the comparison to other relevant sEV cell sources like BM‐MSC and hTERT‐MSC and other functionalities (Louro et al., 2022).

In line with the stronger pro‐angiogenic and anti‐fibrotic effects observed in vitro, ESC‐sEV treatment also improves heart function, as previously reported by Khan et al. (2015) in an in vivo model of permanent MI (Khan et al., 2015). Only mice treated with ESC‐sEV show increased LVEF and FAC 28 days after MI compared to the baseline. Furthermore, adverse remodelling after MI, reflected by increased LVEDV and LVESV, observed in the vehicle‐treated group is not observed after ESC‐sEV treatment. Consistent with these findings, ESC‐sEV treatment shows greater pro‐angiogenic and anti‐fibrotic effects in the MI region of the heart compared to that of mice treated with hTERT‐MSC‐sEV, CPC‐sEV or PBS. Cardiac function improvements conferred by hTERT‐MSC or CPC‐sEV in our in vivo study are milder than those reported in the literature (Q. Li, Xu et al., 2021; Wu et al., 2020). This could be due to differences in factors such as the usage of different in vivo model setups (permanent MI vs. M‐IR, animal model, etc.), sEV concentrations, or the CPC differentiation state.

Notably, in the analysis of differential expression among hTERT‐MSC, ESC, CPC, and their respective sEV, we observe a trend wherein the mRNAs and miRNAs found in both sEV and the parent cells are predominantly enriched within the sEV.This suggests that mRNAs and miRNAs may be selectively sorted into sEV through an active mechanism, which is an active area of research in the EV field (Jeppesen et al., 2023). In line with the greater similarity between ESC and CPC observed in the differential expression analysis and PC analysis, functional enrichment analysis shows fewer canonical pathways with a significantly different NES‐score between ESC‐sEV versus CPC‐sEV than between ESC‐sEV versus hTERT‐MSC‐sEV.

ESC‐sEV consistently display anti‐fibrotic effects in vitro and in vivo. Activated fibroblasts upregulate a core set of pathways including TGF‐β, platelet‐derived growth factor (PDGF), WNT, and Hedgehog (Distler et al., 2019). In addition, increased collagen production and telomere shortening are also processes known to contribute to fibrosis (Distler et al., 2019). Interestingly, the mRNAs and proteins differentially expressed in ESC‐sEV compared to hTERT‐MSC or CPC‐sEV are linked to downregulation of these pathways, including reduced proteins and mRNAs from the TGF‐β and collagen families in ESC‐sEV. Furthermore, certain pro‐fibrotic miRNAs, such as hsa‐miR‐145‐5p, hsa‐miR‐199a‐fp, and hsa‐miR‐223‐3p, are down‐regulated while anti‐fibrotic miRNAs, such as hsa‐miR‐373‐3p, hsa‐miR526b‐3p, hsa‐miR‐448, and hsa‐miR‐194, are up‐regulated (Cheng et al., 2020; Yang et al., 2019).

Our findings also demonstrate that ESC‐sEV exhibit stronger pro‐angiogenic effects than sEV from hTERT‐MSC or CPC in vitro and in vivo. Notably, the functional enrichment analysis indicates that mRNAs and proteins associated with several pathways related to cell growth and angiogenesis, including MYC, telomerase, BDNF, and SLITS and ROBO signalling pathways are consistently up‐regulated in ESC‐sEV compared to hTERT‐MSC and CPC‐sEV (Baudino et al., 2002; Z. L. Liu et al., 2023). Importantly, our analysis of the top differentially expressed mRNAs or protein linked to cell growth and angiogenesis reveals that ESC‐sEV is particularly enriched with fibroblast growth factor‐2 (FGF‐2) protein, which has been previously reported as a pro‐mitotic and pro‐angiogenic factor (Cross & Claesson‐Welsh, 2001; Pasumarthi et al., 1996). FGF‐2 is a prominent constituent of ESC culture media. Our Western blot analysis revealed the presence of FGF‐2 exclusively in ESC‐sEV isolates, and not in pellets obtained from ESC culture media that underwent the same processing as the ESC‐sEVs (Figure S10). Although the precise mechanisms were not investigated, our proteomic data reveals the presence of the FGF‐2 receptor in ESC‐sEVs. This suggests that the high levels of FGF‐2 observed in ESC‐sEVs may, at least in part, result from either active packaging into the vesicles or precipitation when bound to the FGF‐2 receptor during isolation. Additionally, we also observe that the hsa‐miR‐371‐373 cluster and the hsa‐amiR‐486‐5p, known to be associated with cardiomyocyte cell cycle re‐entry and angiogenesis, respectively, are enriched in ESC‐sEV compared to hTERT‐MSC or CPC‐sEV, respectively (Eulalio et al., 2012). While this study focused on the transcriptional profiling of mRNA and miRNA, future investigations could broaden the scope by exploring the expression of additional non‐coding RNAs (ncRNAs), such as lncRNA, circRNA, siRNAs, and piRNAs, in sEV. Non‐coding RNAs are recognized for their varied expression patterns across different cells and various physiological and pathological conditions (Q. Li, Xu et al., 2021). The potential involvement of these sEV biomolecules in cardiac remodelling warrants further exploration.

Obtaining sufficient EV is a major hurdle for translating EV‐based therapies to the clinic (Kennedy et al., 2021). Our study shows that CPC provides the highest yield of sEV per volume of conditioned media, followed by ESC and then hTERT‐MSC, CM, and BM‐MSC. However, it is important to note that optimal growth conditions for both CPC and ESC are under higher cell density compared to that for BM‐MSC or hTERT‐MSC (Foo et al., 2018; Fossett & Khan, 2012). Thus, we also evaluated the number of sEV obtained per viable cell. Our analysis shows that, although hTERT‐MSC, ESC and CPC are the cell types with the higher rate of sEV secretion per number of viable cells, no differences were observed within them.

Our investigation has some limitations that future research should address to further investigate the reparative properties of ESC‐sEV. Among these limitations we would highlight the need to use in vivo models of MI in larger animals, repeated doses of ESC‐sEV post‐MI, treatment in chronic heart failure models, the usage of cell lines from different donors or the usage of engineered ESC‐sEV that incorporate pro‐reparative factors. We did not assess in vivo anti‐inflammatory properties or macrophage polarization influence for the various sEV types due to logistical constraints. This would necessitate a different setup with recurrent doses or shorter timepoints, particularly within the first two to three weeks post‐MI (Pluijmert et al., 2021). Furthermore, the potential clinical use of ESC‐sEV should be considered cautiously. Because pluripotency transcription factors present in ESC can be transferred to their EV (Bobis‐Wozowicz et al., 2015), the concern about potential EV‐associated oncogenic events must be addressed carefully before moving ESC‐sEV into clinical trials and be investigated thoroughly in any future clinical trials that use ESC‐sEV.

In conclusion, we provide evidence that sEV derived from ESC are a promising cell source for inducing cardiac repair and protection after MI. ESC possess characteristics that render them easy to manipulate and genetic engineering, enabling large‐scale production. Moreover, their culture has been extensively evaluated and optimized for defined, xeno‐free culture conditions. Consequently, ESC‐sEV are an interesting option as a cell‐free reparative therapy that warrants further investigations. Future investigations may explore avenues such as genetic engineering of ESC to enhance specific properties of secreted sEV, encompassing aspects like tropism and pro‐regenerative properties. Similarly, in situ modifications of ESC‐sEV composition could be pursued to further tailor their therapeutic potential.

## AUTHOR CONTRIBUTIONS

Hernán González‐King Garibotti contributed to the conceptualization of the project and participated in all the experiments. He carried out the cell culture of VCF, BM‐MSC, hTERT‐MSC, ESC, CPC, and CM, for sEV isolations, and characterized the cells and sEV for functional evaluation. Hernán also performed experiments to evaluate sEV functional properties, including cardioprotection, angiogenesis, cardiac fibrosis, immunomodulation, and proliferation. In addition, he collected sEV samples, performed surgeries and terminations, and conducted histology, plotting data and interpreting results. Hernán also oversaw the bioinformatics analysis, collecting sEV to isolate mRNAs, miRNAs, and proteins, and deciding on data representation and database selection for functional enrichment analysis. He prepared the figures and wrote the manuscript. Patricia G. Rodrigues assisted with the in vivo experiments, including surgeries and echocardiography acquisition and analysis. Tamsin Albery assisted with surgeries in the in vivo experiments. Benyapa Tangruksa and Ramya Gurrapu carried out the data curation and analysis of mRNAs, miRNAs, and protein data. Andreia M. Silva performed RNA and miRNA isolation and quality control, while Kai Liu performed mass spectrometry of protein samples from sEV and their parental cells. Gentian Musa assisted with in vivo experiment terminations and ESC differentiations to CPC. Dominika Kardasz and Bengt Kull carried out the cytokine release by macrophages experiment, and Dominika Kardasz also ran the experiment to evaluate sEV secretion by different types of parental cells. Karin Åvall assisted in vivo experiments, while Katarina Ryden supported histology activities. Tania Incitti and Cecilia Graneli conducted some CPC differentiations, and performed flow cytometry characterization of ESC, CPC, and CM. Nitin Sharma, Jane Synnergen, and Hadi Valadi supported the bioinformatic analysis of OMICs data generated from sEV and parental cells mRNAs, miRNAs, and supported the generation of the transcriptomic datasets. Kasparas Petkevicius, Miguel Carracedo, Sandra Tejedor, and Sepideh Hagvall supported experiments of fibrosis, immunomodulation, and the OMICs analysis. Phillipe Menasché, Niek Dekker, Qing‐Dong Wang, and Karin Jennbacken supervised and conceptualized the project, and contributed to data interpretation.

## CONFLICT OF INTEREST STATEMENT

H.G‐K.G., P.G.R., T.A., R.G., A.S., K.L., G.M., D.K., B.K., K.Å., K.R., T.I., N.S., C.G., A.I., K.P., M.C., S.T., S.H., N.D., Q‐D.W., and K.J., are employees of AstraZeneca and may be AstraZeneca shareholders. The remaining authors declare no competing interests. H. G‐KG, G. M., A. S., K. P., M. C., and A. I. were postdoc fellow of the AstraZeneca R&D postdoc program during this study. D.K. was a member of the AstraZeneca R&D Graduate Program.

## Supporting information

Supplementary Information

## Data Availability

The raw data for each experiment reported can be obtained from the authors upon reasonable request.
