# Supplementary material for: Head‐to‐head comparison of relevant cell sources of small extracellular vesicles for cardiac repair: Superiority of embryonic stem cells
Source: J Extracell Vesicles. 2024 May 6;13(5):e12445. doi: 10.1002/jev2.12445 (PMC11074624; doi:10.1002/jev2.12445)
Supplement: Supplementary file 1 — Supplementary Information [file JEV2-13-e12445-s001.docx]

**Cell Culture**

*Maintenance of human ventricular cardiac fibroblasts (VCF) –* VCF (LONZA; CC-2904) were maintained in FGM^TM^-3 Cardiac Fibroblasts Growth Medium-3 BulletKit^TM^ at 37 ºC, 5% CO_2_. Once the cells reached 80%-90% confluence were dissociated into single cells with TrypLE Express (ThermoFisher Scientific) at 37 ºC for 5 min and seeded at a cell density of 3,500 cells/cm^2^ onto new plates. This cell line was used within passages 1-5.

*Maintenance of human bone marrow derived mesenchymal stromal cells (BM-MSC) –* BM-MSC (LONZA; PT-2501) were maintained in DMEM-low glucose, GlutaMAX^TM^ Supplement, pyruvate medium (ThermoFisher Scientific) supplemented with 10% of Fetal Bovine Serum, mesenchymal stem cell-qualified (ThermoFisher Scientific) and 1% of Penicillin-Streptomycin (ThermoFisher Scientific), at 37 ºC, 5% CO_2_. Once the cells reached 80%-90% confluence they were dissociated into single cells with TrypLE Express (ThermoFisher Scientific) at 37 ºC for 5 min and seeded at a cell density of 5,000 cells/cm^2^ onto new plates. This cell line was used within passages 1-5.

*Maintenance of human adipose tissue derived mesenchymal stromal cells, immortalized by ectopic expression of human telomerase reverse transcriptase (hTERT-MSC) –* hTERT-MSC (ATCC; SCRC-4000 ™) were maintained in DMEM-low glucose, GlutaMAX^TM^ Supplement, pyruvate medium (ThermoFisher Scientific), supplemented with 10% of Fetal Bovine Serum, mesenchymal stem cell-qualified (ThermoFisher Scientific) and 0.2 mg/mL of Geneticin (ThermoFisher Scientific), at 37 ºC, 5% CO_2_. Once the cells reached 80%-90% confluence they were dissociated into single cells with TrypLE Express (ThermoFisher Scientific) at 37 ºC for 5 min and seeded at a cell density of 5,000 cells/cm^2^ onto new plates. This cell line was used between passages 8-25.

*Maintenance of human embryonic stem cell (ESC) Cell Line 121 (SA121) –* ESC (Takara Bio; Y00025*)*  were maintained on Matrigel (Corning) coated plates in mTESR^TM^ 1 medium (STEMCELL technologies) at 37 ºC, 5% CO_2_ according to previously published methods [1]. Fresh mTeSR^TM^ 1 medium was replaced daily and used for sEV isolation. Once the cells reached 80%-90% confluence they were dissociated into single cells with TrypLE Express (ThermoFisher Scientific) at 37 ºC for 5 min and seeded at a cell density of 50,000 cells/cm^2^ onto new Matrigel coated plates in mTESR^TM^ 1 supplemented with 5 µM ROCK inhibitor Y-27632 (Sigma-Aldrich) for the first 24h. This cell line was used within passages 15-35.

*Generation of human ESC derived cardiac progenitor cells (CPC)* - ESC were differentiated to CPC as previously described [1]. *Human embryonic stem cell (ESC) Cell Line 121 (SA121) –* ESC (Takara Bio; Y00025*)* maintained on a Matrigel-coated surface in mTESR^TM^ 1 were dissociated into single cells with TrypLE Express (ThermoFisher Scientific) at 37 ºC for 5 min and were seeded into an evenly distributed monolayer onto a Matrigel-coated cell culture dish at 350,000 cell/cm^2^ in mTESR^TM^ 1 supplemented with 5 µM ROCK inhibitor Y-27632 (day -2) for 24h.

Twenty-four hours later, (day -1), media was changed, and cells were cultured in mTESR^TM^ 1 without ROCK inhibitor Y-27632. On day 0, differentiation was initiated by removing mTESR^TM^1 medium and adding RPMI1640 (ThermoFisher Scientific) supplemented with 1x B27 without insulin (ThermoFisher Scientific) (RPMI/B27-ins) and containing 12 µM GSK3 inhibitor CHIR99021 (Tocris). Media was changed to RPMI/B27-ins 24h later.

On day 3, the medium was changed to the RPMI/B27-ins medium containing 2 µM Wnt-C59 (Tocris), which was changed to basal RPMI/B27-ins on day 5. On day 7, medium was collected for sEV isolation and fresh RPMI/B27-ins medium was added. On day 8, medium was collected and pooled together with day 7 medium for sEV isolation. CPC differentiation was confirmed by flow cytometry analysis of ISl1+/cTNT+/TRA-1-60- expression. sEV isolated from days 7 and 8 conditioned medium were considered as CPC-sEV.

*Generation and maintenance of human ESC derived cardiomyocytes (CM) –* CM were generated from CPC obtained following the CPC progenitor cell differentiation protocol described above. Briefly, on day 8 of CPC differentiation, RPMI/B27-ins medium was substituted for RPMI1640 supplemented with 1x B27 containing insulin (ThermoFisher Scientific), and the cell culture was maintained at 37 ºC, 5% CO_2_ for 15 days, with media change every other day. At day 15 of differentiation the cardiomyocyte state was confirmed by flow cytometry analysis of cTNT. The conditioned media for sEV isolation was collected between days 15 and 25 when performing the media changes.

All cell lines used in this work were tested negative for mycoplasma.

**Preparation of conditioned media before sEV isolation**

Fetal bovine serum (FBS) contains EV [2]. BM-MSC, hTERT-MSC and VCF maintenance media therefore contain FBS-derived EV. For the isolation of sEV, FBS-EVs were first removed from the maintenance medium of each cell source. For this, maintenance medium was transferred to 96 mL quick seal polyallomer tubes (Beckman Coulter) to pellet EVs by ultracentrifugation at 150,000 g for 16 h at 4 ºC using a 45Ti rotor and an Optima XPN ultracentrifuge (Beckman Coulter). Supernatant was then collected and filtered with a pore size of 0.22 µm, as described [2]. This media was then incubated with the cells for 48 h. Subsequently, conditioned FBS-EV-free media was collected and sEV were isolated using a serial ultracentrifugation protocol as described in “sEV isolation” section.

**sEV isolation**

*By serial (ultra)centrifugation*

sEV used in each functional experiment were isolated using a serial ultracentrifugation protocol, as described [2]. Briefly, cell supernatants were centrifuged at 2,500xg for 20 min, and then the supernatant was transferred to 96 mL quick-seal polyallomer tubes (Beckman Coulter) to pellet intermediate-size EVs by centrifugation at 15,000xg for 50 min at 4 ºC using a 45Ti rotor and an Optima XPN ultracentrifuge (Beckman Coulter). Pellets were discarded and supernatants subsequently filtered through a polyethersulfone membrane filter (Sigma-Aldrich) with a pore size of 0.22 µm to remove the remnant intermediate-size EVs. Supernatants were transferred to new tubes and ultracentrifuged at 100,000 g for 120 min at 4 ºC (Type 45 Ti rotor, k-factor 210.4) to pellet sEV. Supernatants were discarded and sEV resuspended in 1 mL of ice-cold phosphate buffered saline (PBS), transferred to a new 96 mL quick seal polyallomer tube, filled with ice-cold PBS and ultracentrifuged again at 100,000xg for 120 min at 4 ºC, as above. Pellets containing sEV were resuspended in 70 – 200 µL of ice-cold PBS, aliquoted and frozen at -80 ºC until further use. An aliquot of each sEV type was thawed and total protein was quantified using Qubit^TM^ (Thermo Fisher Scientific) following manufacturer’s instructions. Then, for every experiment the same amount of total sEV protein within sEV types was used.

*By size-exclusion chromatography*

To evaluate the number of sEV yielded per mL of conditioned media size exclusion chromatography columns from IZON (qEV10) were used. This system allows to reduce handling variability and the differences introduced by the potentially different sedimentation coefficients between cell lines sEV that impact sEV isolation efficiency by ultracentrifugation. Briefly, 70 mL of cell supernatants were centrifuged at 2,500 g for 20 min, the supernatant was transferred to a protein concentrator vessel (ThermoFisher Scientific; Protein Concentrators PES 100kDa) and concentrated by centrifugation at 1,200 g for 7 min at 4 ºC. The concentrated cell supernatant was loaded into the IZON qEV10 Gen 2 35 nm column that was previously prepared in accordance with the manufacturer’s instructions. The fraction of the eluted sample enriched in particles was collected. The particle fraction was further concentrated using a protein concentrator vessel (ThermoFisher Scientific; Protein Concentrators PES 3kDa), taking each sample to the same final volume and then quantified by nanoparticle tracking analysis (NTA) on a NanoSight LM14c (Malvern Panalytical)) instrument. The particles found in the basal medium of each cell type were isolated using the same procedure. Subsequently, these particles were subtracted from the NTA results obtained during the analysis of sEV isolated from the conditioned media of each cell type.

**Transmission electron microscopy of sEV**

Suspensions of sEV were fixed for 10 min in paraformaldehyde 2% (Sigma-Aldrich) in PBS. Carbon-coated 100-mesh copper grids were glow discharged at 7.2 V for 60 s, using a Bal-Tec MED 020 Coating System, and sEV samples were immediately incubated on top of the grids for 15 min, at RT. Grids were then washed in PBS, fixed in glutaraldehyde 1% (Sigma-Aldrich) for 5 min at RT and washed again. sEV adsorbed onto the grids were negatively stained with aqueous uranyl acetate 2% (Sigma-Aldrich) for 2 min, followed by washing, drying and analysis using a FEI Tecnai G2 Spirit transmission electron microscope (ThermoFisher Scientific), equipped with a Morada digital camera (Olympus Soft Image Solutions GmbH).

**Nanoparticle analysis of sEV**

The size distribution and particle concentration of the sEV suspensions were analyzed by nanoparticle tracking analysis (NTA) using a NanoSight LM14c (Malvern Panalytical) instrument, equipped with a blue laser (488 nm, 70 mW) and CMOS camera (Hamamatsu Photonics). 1.73 µg of sEV protein were diluted in 1 mL of PBS for measurement of 20-100 particles/frame in all conditions, injected at a speed of 100 a. u. into the measuring chamber and sEV flow recorded in triplicate measurements of 90 s each, at 25 ºC. Equipment settings for data acquisitions were kept constant between measurements with camera level set to 14, auto settings off, screen gain set to one and threshold set to seven. Data analysis was performed with NTA 3.2 software (Malvern Panalytical). Background particles originating from the corresponding buffer were subtracted during the analysis.

**Western blotting analysis of sEV and cell lysates**

Cell cultures were trypsinized and, centrifuged at 300xg for 7 min. Cell pellets were washed with ice-cold PBS and centrifuged again at 300xg for 7 min. Cell pellets were then lysed with Pierce RIPA buffer (ThermoFisher Scientific) supplemented with complete EDTA-free protease inhibitor cocktail (Sigma-Aldrich) for 30 min, on ice. Lysates were centrifuged at 14,000 rpm for 10 min at 4 ºC, supernatant recovered and preserved at -20 ºC. Protein content of sEV suspensions and parental cell lysates was quantified using Qubit protein assay kit (ThermoFisher Scientific), according to the manufacturer’s instructions. Five μg of total protein were loaded on SDS-PAGE gels for each sample to analyze the exosome markers by Western blot, whereas, due to the low protein concentration of the basal medium pellet, 0.5 μg of total protein were used to investigate the presence of FGF-2. All samples were prepared in NuPAGE LDS sample buffer (ThermoFisher Scientific). For the detection of CD63 and CD81, Western blot was carried out in non-reducing conditions. For the detection of all the other proteins, Western blot was carried out in reducing conditions by supplementation of NuPAGE LDS sample buffer with NuPAGE Reducing Agent (ThermoFisher Scientific). Then, protein samples were denatured at 75 ºC for 10 min and resolved on NuPAGE 4-12% Bis-tris gels (ThermoFisher Scientific) in NuPAGE MES SDS running buffer (ThermoFisher Scientific). Proteins were transferred onto PVDF membranes (Bio-Rad Laboratories), using a Trans-Blot Turbo Transfer System (Bio-Rad Laboratories). Membranes were blocked with Odyssey tris-buffered saline (TBS) buffer (Li-COR) for 1 h at RT and incubated overnight at 4 ºC with the following primary antibodies: mouse monoclonal anti-CD63 (ab59479, Abcam), mouse monoclonal anti-CD81 (ab79559, Abcam) and rabbit polyclonal anti-CALNEXIN (ab22595, Abcam), anti-ALIX (ab275377), anti-TSG101 (ab125011), anti-FGF-2 (Cell Signaling Technology®; #20102) all diluted 1:1,000 in Odyssey TBS buffer. Membranes were then washed three times with TBS.Tween-20 0.05% (TBS-T; Sigma-Aldrich) and incubated for 1 h at RT with anti-mouse or anti-rabbit fluorophore conjugated secondary antibodies (all from LI-COR), diluted 1:20,000 in TBS-T. Membranes were imaged on an Odyssey CLx imaging system (LI-COR) equipped with Image Studio v4.0 software.

**RNA extraction from sEV and cell lysates**

Cell pellets containing 1x10^6^ cells were prepared as above and, after washing, lysed with 500 µL QIAzol lysis reagent (Qiagen). sEV samples were diluted in PBS to a final volume of at least 100 µL and mixed with QIAzol lysis reagent at a 1:3 ratio (vol/vol). Total RNA was then extracted using the miRNeasy Micro kit (Qiagen), with on-column contaminant DNA digestion, following the manufacturer’s protocol for samples with less than 1 µg RNA. RNA isolated and was quantified by spectrometry using Qubit RNA assay kit (ThermoFisher Scientific) according to manufacturer’s instructions.

***In vitro* functional studies**

*Angiogenesis assays*

*Tube formation assay –* human cardiac microvascular endothelial cells (hCMVE-C; LONZA; CC-7030) were stained with CellTracker (ThermoFisher Scientific). Then, a total of 3 X 10^5^/cm^2^ hCMVE-C were seeded into each well of a 96-well plate pre-coated with 50 µL of growth factor-reduced Matrigel (Corning). For experimental treatments, hCMVE-C were incubated in EBM-2 medium and treated with 10 µg/mL of sEV total protein. Cells were then incubated 12 h with the different treatments to evaluate formation of capillary tube-like structures [3]. As a negative control, hCMVE-C were incubated in EBM-2 without supplements or FBS and as a positive control cell were incubated either in EGM-2 Bulletkit complete medium or EBM-2 plus FGF-2 50 ng/mL (ThermoFisher Scientific). Images were captured from the center of the well from three different samples at a magnification of 10X using an inverted microscope (Leica DM6000, Leica Microsystems) and the number of loops produced by hCMVE-C quantified.

*hCMVE-C proliferation assay –* A total of 6 X 10^3^ hCMVE-C were seeded in each well of a 96-well plate in standard culture conditions. For experimental treatments 24 hours later, hCMVE-C EGM-2 media was substituted for EBM-2 medium and treated with 10 µg/mL of total sEV protein of each sEV type separately. As a negative control, hCMVE-C cells were incubated in EBM-2 without supplements or FBS and as positive control cells were incubated in EGM-2 BulletKit complete medium or VEGF 6 ng/mL. Twenty-four hours later the cells were washed with PBS, and plasma membranes were stained with 1X CellMask Deep Red (ThermoFisher Scientific) at 37 ºC for 15 min. Cells were fixed in 4% paraformaldehyde for 15 min at RT, permeabilized and blocked with PBS/0.1% Triton-X-100/10% FBS for 30 min at RT. Cells were incubated with KI67 primary antibody (Abcam; ab15580) at 4 ºC overnight, followed by three washes in PBS, prior to incubation with secondary antibodies – goat anti-rabbit IgG Alexa Fluor 488 (ThermoFisher Scientific). Nuclei were counterstained with Hoechst 33342 (5 µg/mL, Invitrogen). Images were acquired at a magnification of 10X using ImageXpress Micro Confocal High-Content Imaging System (Molecular Devices) and analyzed with MetaXpress High-Content Image Acquisition and Analysis Software (Molecular Devices).

*Aortic ring assay –* The aortic ring assay was performed as described [4]. Aorta from 12-week-old male C57BL/6Ncrl mice were collected. Fat tissue was removed from the aorta using forceps and blood remaining inside was washed out by flushing with Opti-MEM^TM^ (ThermoFisher Scientific) supplemented with 2.5% of FBS (ThermoFisher Scientific) and 1% of Penicillin-Streptomycin (P/S; ThermoFisher Scientific). Then, the aorta was sliced into 0.5 mm rings and serum-starved overnight in Opti-MEM supplemented with 1% P/S. Rings from different aortas were kept separated. The following day, aortic rings from different animals were randomized and embedded individually in 50 µL of growth factor-reduced Matrigel (Corning) on 96-well plates. After aortic rings embedding, Opti-MEM medium supplemented with 2.5% FBS and 1% P/S, and containing the different treatments was added to every well. A concentration of 10 ug/mL of sEV total protein was used for sEV treatments. Human FGF-basic (FGF-2/bFGF) Recombinant Protein (ThermoFisher Scientific) at 20 ng/mL was used as a positive control and sEV vehicle, PBS, was used as negative control. Maintenance media and treatments were refreshed every other day for 12 days. After 12 days, aortic rings were fixed with 4% paraformaldehyde and stained for α-SMA (Dako, M0851) and CD31 (R&D Systems, AF3628) as described [4]. Images and stacks were acquired using Zeiss LSM 980 confocal microscope. Image processing and analysis was performed using Imaris software (Oxford Instruments Group) and ImageJ.

*Cardiac fibrosis assay*

A total of 3 X 10^3^ VCF were seeded into each well of a 96-well plate and serum-starved for 24 h. Then, TGF-β1, DxS and L-ascorbic acid (pro-fibrotic cocktail) were added to the media to induce fibrosis for 2 days as described [5]. To evaluate the anti-fibrotic potential of the different sEV preparations, the amount of each sEV type containing 10 µg/mL of total protein was added at the same moment than the pro-fibrotic cocktail. Two days after starting the treatment, cells were washed with PBS, and plasma membranes were stained with 1X CellMask Deep Red (ThermoFisher Scientific) at 37 ºC for 15 min. Cells were fixed in 4% PFA for 15 min at RT, permeabilized and blocked with PBS/0.2% Triton-X-100/10% FBS for 30 min at RT. Cells were incubated with primary antibodies (mouse anti-human α-SMA, Dako, M0851, 1:100; Rabbit anti-human α-Collagen 1α1 telopeptide, Rockland, 600-401-D20) at 4 ºC overnight, followed by three washes in PBS. Cells were then incubated with the secondary antibody’s goat anti-rabbit IgG Alexa Fluor 488 or goat anti-mouse IgG Alexa Fluor 594 (1:500, Life Technologies), for 1 h at RT, followed by three washes in PBS. Nuclei were counterstained with Hoechst 33342 (5 µg/mL, Invitrogen). Images were acquired at a magnification of 10X using ImageXpress Micro Confocal High-Content Imaging System (Molecular Devices) and analyzed with MetaXpress High-Content Image Acquisition and Analysis Software (Molecular Devices).

*Human blood samples*

All donors provided informed written consent for blood donation as approved by AstraZeneca’s Institutional review board and local ethic committee (033–10). Heparin-anticoagulated whole blood samples were collected from healthy donors and processed on the same day of collection.

*Macrophage polarization assay*

Human peripheral blood mononuclear cells (PBMC) were prepared from blood samples from healthy donors using Ficoll gradient (Eurobio) centrifugation. Freshly collected blood samples were used for each experiment.

PBMC from healthy donors were suspended at a concentration of 100x10^6^ cells/mL in Monocyte Attachment Medium (Promocell) and seeded at a density of 1.5 x10^6^ viable cells/cm^2^. On Day 0, monocytes were incubated for 90 min at 5% CO_2_ and 37 ºC to allow adherence. Non-adherent cells were discarded by vigorously swirling the culture and aspirating the supernatant. Adherent cells were then washed and the appropriate amount of complete M0, M1, M2 Macrophage Generation Medium (Promocell) was added to the cells for 6 days. Under these conditions, monocytes differentiate into M1-like or M2-like polarized macrophages. After 6 days, the culture medium was replaced with fresh complete M0, M1, M2 Macrophage Generation Medium DXF with or without sEV (10 μg/mL of total sEV protein per well). Macrophages were then incubated for another 2 days at 37 ºC. At day 8, the culture medium was supplemented with interferon (IFN)-γ (50 ng/mL) and lipopolysaccharide (LPS; 10 ng/mL) for M1 macrophage polarization, with 20 ng/mL IL-4 and 20 ng/mg IL-13 for M2 macrophage polarization (all from ThermoFisher Scientific) or with macrophage colony-stimulating factor (M-CSF) medium for M0 macrophages. Each of these conditions were exposed or not to sEV (10 µg/mL). The next day (day 9), cells were detached with the Macrophage Detachment Solution DXF (Promocell) for 45 min at RT. Monocytes and macrophages were analyzed at day 9 of culture, after polarization and exposure to EV, as described above. They were suspended in MACSBuffer (Miltenyi), centrifuged for 10 min at 500 g, counted and labelled at 200,000 cells per condition with anti-CD86-FITC, anti-CD80-APC-H7, anti-CD163-BV421 and anti-CD206-PE antibodies (all from BD Bioscience). All antibodies were used at a dilution of 1:100. After incubation, cells were washed and suspended in 200 µL of MACS Buffer for FACS analysis. Samples were analysed on a Fortessa X20 flow cytometer (BD Bioscences); results were analyzed with FlowJo software (FlowJo V10.4.2, FlowJo LLC) and the percentage of the different populations compared to the total cell population were represented normalized to the vehicle treatment (PBS).

An aliquot of the media from the macrophages at day 9 under the different treatments was collected to evaluate the concentration of IL6 and TNF-α using MESO SECTOR S 600MM (Meso Scale Discovery) instrument.

Experiments were performed three independent times with three different donors.

*Cardiomyocyte proliferation assay*

iPSC derived cardiomyocytes (iCell® cardiomyocytes, Cellular Dynamics International) were thawed and seeded in iCell Plating Medium according to manufacturer’s instructions (iCell®) at a cell density of 60,000 cells/cm^2^ in a 384-well black plate, optically clear polymer bottom (Corning). The following day plating media was switched to maintenance medium which contains William’s E Medium (ThermoFisher Scientific) supplemented with Cocktail B (ThermoFisher Scientific). Maintenance medium was changed every other day. The experiment started on day 6 after thawing the cells. At day 6, EdU was added at 2 µM together with the different treatments. sEV samples were added at a concentration of 10 ug/mL of sEV total protein. sEV vehicle, PBS, was used as negative control and Human FGF-basic (FGF-2/bFGF) Recombinant Protein (ThermoFisher Scientific) at 20 ng/mL was used as a positive control. After treatments addition, the cells were incubated at 37 ºC and 5% CO_2_ during 48 h. Then cardiomyocytes were fixed with 4% PFA and cardiomyocytes cell cycle activity was detected following Click-iT^TM^ EdU Cell Proliferation Kit (ThermoFisher Scientific) manufacturer’s instructions. Images were acquired at a magnification of 4X using ImageXpress Micro Confocal High-Content Imaging System (Molecular Devices) and analyzed with MetaXpress High-Content Image Acquisition and Analysis Software (Molecular Devices).

*Cardioprotection assay*

iPSC derived cardiomyocytes (iCell® cardiomyocytes, Cellular Dynamics International) were thawed and seeded in iCell Cardiomyocytes Plating Medium according to manufacturer’s instructions (iCell®) at a cell density of 60,000 cells/cm^2^ in a 384-well black plate, optically clear polymer bottom (Corning). The following day plating media was switched to iCell Cardiomyocytes Maintenance medium. Maintenance medium was changed every other day. Two weeks after thawing, cells were washed twice with glucose deprived medium (DMEM) and iCell Maintenance Medium was switched to Glucose deprived medium containing 0.1 % P/S either together with sEV from the different cell sources or vehicle (PBS), and placed in an incubator at 37 ºC, 5% CO_2_ and 3% O_2_. Six hours later, hiPSC-CM nuclei were stained with Hoechst 33342 (5 µg/mL, Invitrogen) and apoptotic nuclei were detected by staining them with 1 µg/mL of eBioscience^TM^ Propidium Iodide (ThermoFisher Scientific). Images were acquired at a magnification of 4X using ImageXpress Micro Confocal High-Content Imaging System (Molecular Devices) and analyzed with MetaXpress High-Content Image Acquisition and Analysis Software (Molecular Devices).

***In vivo* studies**

*Cardiac ischemia and reperfusion injury in mice*

Animal work was performed in accordance with the National Institute of Health (NIH) guidelines for use of experimental animals and the study protocol was approved by the Animal Ethics Committee at Gothenburg University (Gothenburg Ethical Review Board number EA (1173-2017). The Male immune-competent C57BL/6Ncrl mice at 10-12 weeks of age (Charles River) and weight of ~25 g were housed on a 12 h light/12 h dark cycle, ambient temperature at 21 – 22 ᵒC and 50 % humidity. For induction of transitory LAD occlusion, the animals were anesthetized with 2 – 3 % Isoflurane (Forene ®) mixed with oxygen, intubated, and connected to a mice ventilator. The mice were ventilated with air ~800 mL/min and oxygen ~100 mL/min (~230 strokes/min [MiniVent Ventilator for Mice (Model 845), Harvard Apparatus]). Core temperature was continuously monitored and maintained at 35 - 36.5 ᵒC by a heating operating table and heating lamp controlled by rectal thermometer. Electrodes were inserted under skin to register and monitor electrocardiography (ECG; PharmLab). The mice were subjected to a left thoracotomy at the fourth intercostal space ~2 to 3 mm to the left of the sternum. A rib spreader was used to keep the incision open. The pericardium was opened, the left anterior descending coronary (LAD) was localized, and an 8-0 suture (Braun) was used to temporally occlude it. Fifty-five min after LAD occlusion the suture was removed to reperfuse the muscle and 45 µL of the corresponding treatment was intramyocardially injected in the border zone of the infarcted ventricle using an Insulin syringe (Becton, Dickinson and Company). The chest was then closed, and the mice were monitored during continued maintenance of body temperature and ventilation until they regained consciousness and could be disconnected.

The animals were randomized into 4 treatment groups where the sEV dosing regimen was guided by a comprehensive review of existing literature [3, 6, 7]: vehicle (PBS; 13 animals), 20 µg protein of hTERT-MSC-sEV (10 animals), ESC-sEV (10 animals) or CPC-sEV (10 animals) . The animals were euthanized the day after day 28 echocardiographic examination. For histologic examination of the heart, the organ was fixed in 4% buffered formalin, followed by paraffin embedding. The investigators were blinded to experimental settings during all data acquisition and analysis.

*Electrocardiography*

ECG recordings were acquired during MI surgery using tree-lead subdermal needle electrodes, connected to a PowerLab 8/30 data acquisition device (model ML870, ADInstruments), and an animal Bio Amp biological potential amplifier (model ML136, ADInstruments) as previously reported [8]. RR-, PR-, QRS- and QT- intervals, P-duration, P-, Q-, R- S- and T-amplitudes, ST-height and heart rate were analyzed using the ECG Analysis module in the Lab-Chart Pro.

*Echocardiography*

Echocardiography to assess heart function was performed as described 24 h and 28 days after cardiac LAD ischemia reperfusion injury [9]. Mice were anaesthetized using 2.5% isoflurane (Forene ®) mixed with air and then maintained on 1.5 – 2% isoflurane during the assessment. During the examination, the mice were kept on a Physio Plate (Visualsonics) to keep normal body temperature and to monitor ECG and respiration. The ultrasound probe (MS400, 18-38 MHz) was connected to an ultrasound biomicroscope (Vevo 2100 System, Visualsonics). ALV long-axis parasternal B-mode view was captured, followed by a 90º clockwise rotation of the ultrasound probe adjusted to obtain short axis B-mode views at the following three levels: (i) at level of left ventricle (LV) base, (ii) at an intermediate position, approximately at the level of the papillary muscles, and (iii) at the apex level. From these acquisitions, a 3D reconstruction of the LV geometry and the calculation of LV end-diastolic volume (LVEDV) and LV end-systolic volume (LVESV), ejection fraction (EF), fractional shortening (FS), and fractional area change (FAC) were possible using the modified Simpson’s method (Vevo Lab 5.6.1, VisualSonics) [10].

All measurements were averaged from three consecutive cardiac cycles with animal’s treatment group blinded to the sonographer.

**Histology**

For histologic examination the heart excised hearts were directly preserved in 10% neutral buffered formalin for fixation in 2 days. Hearts were transversely sliced in 1 mm slices dehydrated and embedded in paraffin (Magnus Tissue Processor; Milestone Medical). 4µm sections were cut and stained with Hematoxylin-Eosin and Masson’s trichrome stain.

Immunohistochemistry was carried out according to manufacturer’s recommendation and all reagents except antibodies were Ventana products (Roche). Antigen retrieval was done in Ventana Cell Conditioner 1 for 24 min at 95 °C. Anti-CD31 primary antibody (ab28364, Abcam) was added for 1 hour at 37°C followed by secondary anti-rabbit oMap anti-rabbit- HRP, with DAB chromogenic detection. Next, images of the heart slices were taken in PANNORAMIC SCAN II (3D HISTECH). Infarct size was measured using ImageJ in the heart’s slices stained with Masson’s trichrome stain.

**Proteomics**

Protein digestion was conducted on recovered sEV and parental cells containing an equal amount of protein. Briefly, samples are denatured and reduced using a 30 min one-step solution of 8 M urea (#U1250, Merck) and TCEP bond-breaker solution (#77720, Thermo Fisher Scientific), followed by a 30 min alkylation step using a 2-chloroacetamide reagent (#22790, Merck). Protein was digested overnight in trypsin (#EMS0004, Merck) and ceased by the addition of formic acid. Resulting digests were analyzed using a Q-Exactive HF mass spectrometer (ThermoFisher Scientific) coupled with an Evosep One (Evosep) automatic sample loader equipped with Evotip disposable C18 trap columns (Evosep) for in-line peptide desalting and purification. Peptides were separated on an 8 cm analytical reverse-phase column (Evosep) with gradient off-set focusing to achieve a 3%-40% acetonitrile within a 22 min loop (60 samples/day scheme) at a 0.5 µL/min flow rate.

MS raw files were analyzed by MaxQuant software (v1.6.15.0). Proteins were identified using the Uniprot FASTA database (H. sapiens, June 2019, UP000005640) with N-terminal acetylation and methionine oxidations as variable modifications, and cysteine carbamidomethylating as a fixed modification. The false discovery rate (FDR) was set to 1% by reverse database search for both proteins and peptides with a minimum length of 7 amino acids. Enzyme specificity was set as trypsin (cleavage at C-terminal to arginine and lysine). A maximum of 2 missed cleavages were allowed in the database search. Peptide identification was performed with an initial precursor mass deviation tolerance up to 6 ppm and a main mass deviation tolerance to 20 ppm. Matching between runs was performed among samples. Proteins matching to the reversed database were filtered out. For protein quantification, MaxQuant computes raw protein intensities as the sum of all identified peptide intensities. Label-free quantification (LFQ) and intensity-based absolute quantification (iBAQ) were calibrated from raw protein intensities with a minimum peptide ratio count of 1.

**Proteomics data processing**

The steps prior to protein data analysis included data collection and data quality control (QC) [11]. The MaxQuant protein abundance measurements were acquired in tab-separated file and loaded to R. The data has a total of 6 samples with 3 biological replicates each. These 18 columns possess the intensity data which reflect the protein abundances. The QC steps included removal of false positives such as potential contaminants, reverse proteins and the ones only identified by site. Q-values indicate the true positive protein identifications and thus check for q-value 0.01 as a threshold was performed [12].

Duplicate gene symbols were handled by addition of their corresponding protein identifiers to the gene symbols separated by hyphen. Processing was performed using Differential Enrichment analysis of Proteomics data (DEP) version 1.10 [13], an R/Bioconductor package, that provides integrated mass spectrometry proteomics data for differential protein expression or differential enrichment. The data was normalized using variance stabilizing normalization and missing data imputation was performed using the k-nearest neighbor approach using DEP package. The differentially expressed proteins with p-value < 0.05 were considered as statistically significant. The volcano plots were generated using Enhanced Volcano R package (version 1.16) with log fold change cut-off set to ± 0.5 and p-value < 0.05.

**RNA-Seq Analysis**

The mRNA-Seq data was generated and processed by the SciLifeLab National Genomics Infrastructure at the Uppsala Multidisciplinary Center for Advanced Computational Science. The library construction was performed using TakaraSMARTer Stranded Total RNA-Seq, Pico Input Mammalian V3 kit, which was designed for very low input total RNA samples. The samples were sequenced on NovaSeq6000 (NovaSeq Control Software 1.7.5/RTA v3.4.4) with a 151nt (Read1) -19nt (Index1) -10nt (Index2) -151nt (Read2) setup using 'NovaSeqXp' workflow in 'S4' mode flowcell. For miRNA-Seq, the library construction was performed using QIAseq miRNA low input method. The miRNA samples were sequenced on NextSeq2000 (NextSeq 1000/2000 Control Software 1.4.1.39716/RTA 3.9.25) with a 101nt (Read1) -8nt (Index1) setup using 'P2' flowcell. For both the mRNA- and miRNA-Seq data, the Bcl to FastQ conversion was performed using bcl2fastq_v2.20.0.422 from the CASAVA software suite. The quality scale used is Sanger / phred33 / Illumina 1.8+.

Processing of FASTQ files was carried out as follows. The sequenced reads were quality controlled with the FastQC software and pre-processed with Trim Galore. The processed reads were then aligned to the reference genome of Homo sapiens (build GRCh38) with the STAR aligner and Bowtie1 for mRNA and miRNA, respectively. Read counts for genes and normalized TPM values were calculated. Technical documentation on the RNA-seq pipeline for the mRNA and miRNA samples can be accessed here: https://github.com/nf-core/rnaseq and https://github.com/nf-core/smrnaseq.

**Differential expression analysis of the transcriptomic data**

The raw mRNA gene count data contained 60,669 transcripts, 5 replicates each from ESC, ESC-sEV, hTERT-MSC, hTERT-MSC-sEV, CPC, and CPC-sEV. The raw miRNA count data contained 2,056 transcripts including 5 ESC samples, 5 ESC-sEV samples, 2 hTERT-MSC samples, 5 hTERT-MSC-sEV samples, 5 CPC samples, and 4 CPC-sEV samples. The data were imported into R for bioinformatic analysis. The genes with no counts were filtered and the data was normalized using the VarianceStabilizingTransformation() function for mRNA gene count data and using the rlog() function for the miRNA gene count data. To visualize the clusters and the reproducibility of the samples, PCA plots were generated for the top 1,000 most variable genes using the plotPCA() function.

The statistical testing for differential expression was carried out using the DESeq2 R-package [14]. Filtering and normalization of the raw counts were performed for sEV of each cell line separately using the DESeq() function in the DESeq2 package. The Wald test was used for the identification of differentially expressed genes (DEGs). P-values were adjusted for multiple testing using the Benjamin Hoch method and an adjusted p-value (adjP) of ≤ 0.05 was considered statistically significant. A log2 fold change (log2FC) shrinkage was carried out using ashr shrinkage estimator to reduce the variability of the low expressed genes. A result table with log2FC, p-values and adjusted p-values (adjP) was generated. Genes with adjP ≤ 0.05 and |log2FC| > 2.5 are considered significant and are used for downstream functional and pathway analysis. The contrasts for both mRNA- and miRNA-Seq data used for the differential expression analysis were as follows: 1) ESC-sEV vs hTERT-MSC-sEV, 2) ESC-sEV vs CPC-sEV, 3) ESC-sEV vs ESC, 4) hTERT-MSC-sEV vs hTERT-MSC and 5) CPC-sEV vs CPC. The DEGs and differentially expressed miRNA (DE–miRNA) were visualised in volcano plots generated using the EnhancedVolcano R-package.

**DE-miRNA target prediction**

miRWalk and MicroRNA Target Filter function from Ingenuity Pathway Analysis (IPA) software were used to predict the target mRNA of DE-miRNA and to investigate the correlation between miRNA and mRNA and to analyse the potential role of these miRNA [15, 16]. The predicted targets from MirWalk were filtered to only include validated targets with binding position on the 3´ ends of the UTRs of the targets and have entry on miRTarBase, miRDB, and Targetscans. The predicted targets from IPA were filtered to include only the targets that were experimentally observed. The results from both software were combined. The predicted DE-miRNA targets were then compared to the DEGs and input for subsequent pathway analysis using IPA.

**Functional enrichment analysis of the differentially expressed mRNAs and proteins**

Functional enrichment analyses were performed of the differentially expressed mRNAs and proteins were performed using the fgsea package version 1.24 in R and significantly regulated pathways was identified. For this analysis 3,090 Canonical Pathway (CP) gene sets were downloaded from the Molecular Signatures Database (MSigDB) [17]. The identified enriched pathways were manually classified into cell growth and angiogenesis, fibrosis, or immunomodulatory related pathways based on literature annotation and visualized in a heatmap. Is important to note that this classification is made to facilitate the interpretation of the data, but these pathways may participate in many other processes. Top 5 up- and down-regulated mRNAs and proteins that are identified as regulators of these categories were extracted for further exploration.

**Functional enrichment analysis of the differentially expressed miRNAs**

To further explore the functions of the filtered DE-miRNA, their target genes were input to the IPA software [15]. The analysis was carried out using the Core- and Comparison-Analysis functions. The list of top canonical pathways, associated to the expression of DE-miRNA targets were obtained. IPA displays significant canonical pathways with p-values calculated by Right-Tailed Fisher’s Exact Test, where P ≤ 0.05 was deemed statistically significant. The identified enriched pathways were manually classified into cell growth and angiogenesis, fibrosis or immunomodulatory related pathways based on literature annotation and visualized in a heatmap. Is important to note that this classification is made to facilitate the interpretation of the data, but these pathways may participate in many other processes. The top 5 up- and down-regulated miRNAs that are identified as regulators of these categories were extracted for further exploration.

**Expression visualisation**

The baseMean expression output from *DESeq2* of DEGs, DE-miRNA, and DE-proteins (P < 0.05) were plotted against P value or adjusted P value using *ggplot2* R package to visualise whether there is a trend between highly expressed molecules to the differential expression significance. BaseMean is the average of the normalized count values, dividing by size factors, taken over all samples. The base mean was log_10_ transformed while the p values were -log_10_ transformed before plotting to remove the skewness of the data and improve the visualisation of the differences that are clustering around 0.

Several genes/miRNAs/proteins of interest that are differentially expressed (ESC-sEV compared to hTERT-MSC-sEV and CPC-sEV) and are associated with immunomodulation, fibrosis, cell growth and angiogenesis, were highlighted on the scatter plots and their expressions were visualised on box plots together with some known housekeeping genes as reference. The normalised expression values used for the boxplots include normalised abundance for proteins, variance stabilization transformation (VST) for genes, and regularized log transformation (rlog) for miRNAs. The housekeeping molecules and EV markers, including ACTB, CD9, CD81, LAMP1, GAPDH, and PDCD6IP were plotted to compare their expression to those of the genes and proteins of interest. The agreement on a particular housekeeping miRNA for miRNA plotting is limited.

**Statistical analysis and reproducibility**

Data are presented as means ± SE. Differences between two groups were evaluated for significance with Student’s t-test, and differences among three or more groups were evaluated via one-way analysis of variance (ANOVA) followed by Dunnett for comparisons to the same sample and Tukey’s or two-stage-step-up method of Benjamini, Krieger and Yekutieli [18], for multiple comparisons post-hoc tests. Where more than one factor influenced the variable being measured, two-way ANOVA was used to test for a significant effect of each factor as well as an interaction between factors, followed by two-stage-step-up method of Benjamini, Krieger and Yekutieli post-hoc test. Statistical significance was assigned at p<0.05, and statistical difference levels were assigned as follows in the figures, *p<0.05, **p<0.01, ***p<0.001, ****p<0.0001. Statistical tests used are also stated in the legend. All statistical tests and graphs were generated using GraphPad Prism 9 software. All presented animal experiments were performed once.

Combinations of at least three independent cell cultures were used to isolate sEV for every experiment. The sEV from the different isolations were combined to test their effects in the different functional experiments. Every experiment was performed at least three times in duplicates.

**Data availability**

All relevant data are available from the corresponding author upon reasonable request.

OMICS raw and processed data are available for download at Gene Expression Omnibus (https://www.ncbi.nlm.nih.gov/geo/).

**Supplementary figures**

**
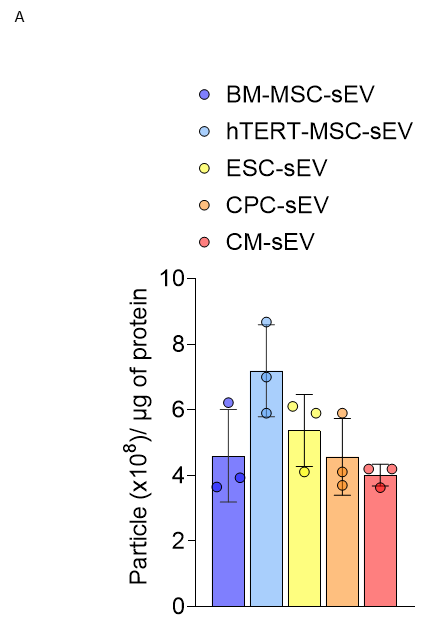
**

**Supplementary Figure 1. Nano-particle concentration per microgram of total sEV protein across the different sEV types. A:** Nano-particles quantified by NanoSight NS300 per μg of total sEV protein quantified with Qubit^TM^ (Thermo Fisher Scientific). *P<0.05; **P<0.01; ***P<0.001. One-way ANOVA multiple comparisons analysis followed by Tukey’s post-hoc test were applied. Dots in the bar graphs represent biological replicates (N). N=3 in each experiment.


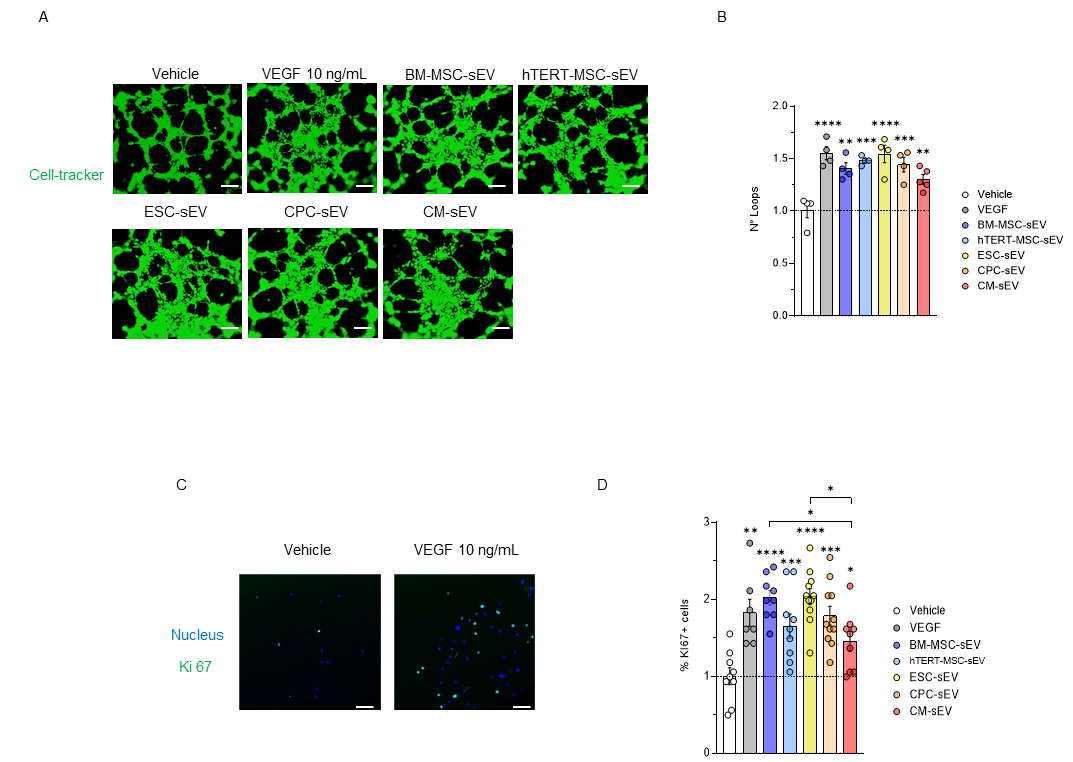


**Supplementary Figure 2. sEV from BM-MSC, hTERT-MSC, ESC, CPC, and CM, promote proliferation and tube formation of human cardiac coronary microvascular endothelial cells. A:** Fluorescence microscopy pictures of human cardiac coronary microvascular endothelial cells (hCMVE-C; stained green with CellTracker Green) seeded on top of Matrigel and treated with vehicle, 10 ng/mL of VEGF or the indicated type of SEV. In the representative pictures is possible to observe the tubes formed on the Matrigel. Scale bars: 100 µm. **B:** Quantification of the number of loops produced in the Matrigel by the hCMVE-C in response to indicated treatments. **C:** Fluorescence microscopy pictures of hCMVE-C. Nuclei are stained with Hoechst (blue) and primary antibodies against KI67 together with Alexa 488 labeled secondary antibodies (green) are used to detect nuclei of proliferating cells. Scale bars: 100 µm. **D:** Quantification of the percentage of hCMVE-C nuclei that are positive for KI67 in response to indicated treatments. *P<0.05; **P<0.01; ***P<0.001. One-way ANOVA multiple comparisons analysis followed by Tukey’s post-hoc test were applied. Dots in the bar graphs represent technical replicates (n). N=3 in each experiment.


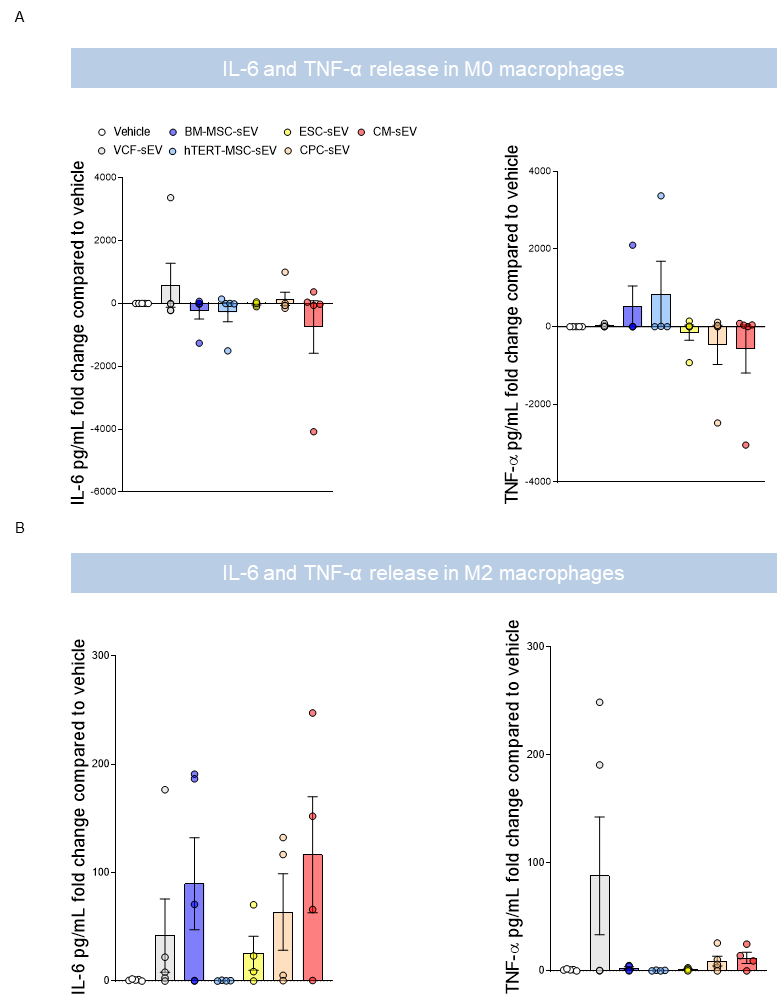


**Supplementary Figure 3. sEV from VCF, BM-MSC, hTERT-MSC, ESC, CPC or CM do not induce significant changes in IL-6 or TNF-α secretion from M0 or M2 macrophages. A:** IL-6 and TNF-α secreted from M0 macrophages treated with different types of sEV. **B:** IL-6 and TNF-α secreted from M2 macrophages treated with different types of sEV. One-way ANOVA multiple comparisons analysis followed by two-stage-step-up method of Benjamini, Krieger and Yekutieli post-hoc test were applied. Dots in the bar graphs represent technical replicates (n). N=3 in duplicates.


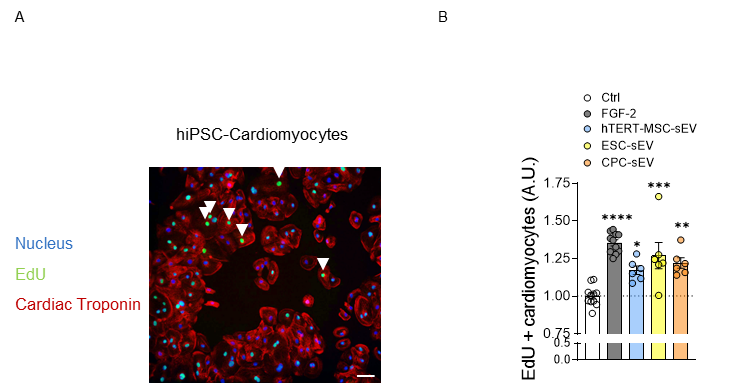


**Supplementary Figure 4. Measuring effects of top sEV candidates on an *in vitro* model of hiPSC-CM proliferation. A:** Fluorescence microscopy pictures of human cardiomyocytes derived from induced pluripotent stem cells (hiPSC-CM). EdU (green) stains nucleus of cardiomyocytes that have entered the cell cycle to undergo cell division. Cardiac troponin was stained using anti-cTNT antibodies to label cardiomyocytes (red). Hoechst (blue) was used to stain the nucleus of cardiomyocytes. **B:** Quantification of the percentage of cardiomyocytes that are positive for EdU incorporation after treatment with or without sEV from hTERT-MSC, ESC or CPC. *P<0.05; **P<0.01; ***P<0.001. One-way ANOVA multiple comparisons analysis followed by Tukey’s post-hoc test were applied. Dots in the bar graph represent technical replicates (n). N=3.


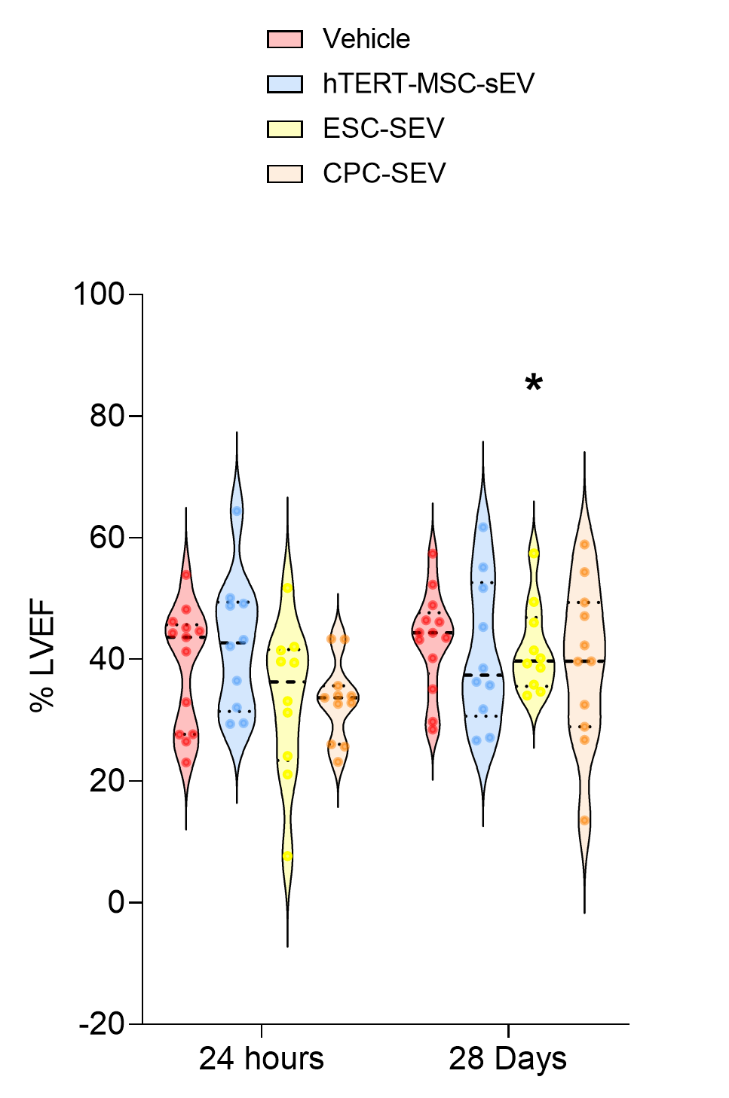


**Supplementary Figure 5. ESC-sEV increase % of LVEF after MI. A:** Graph showing the % of LVEF in infarcted hearts after vehicle or ESC, hTERT-MSC, CPC sEV treatment. *P<0.05; **P<0.01; ***P<0.001. A two-way ANOVA mixed effects analysis followed by two-stage-step-up method of Benjamini, Krieger and Yekutieli post-hoc test were applied[18]. N=13 in vehicle group and N=10 on sEV treatments groups.


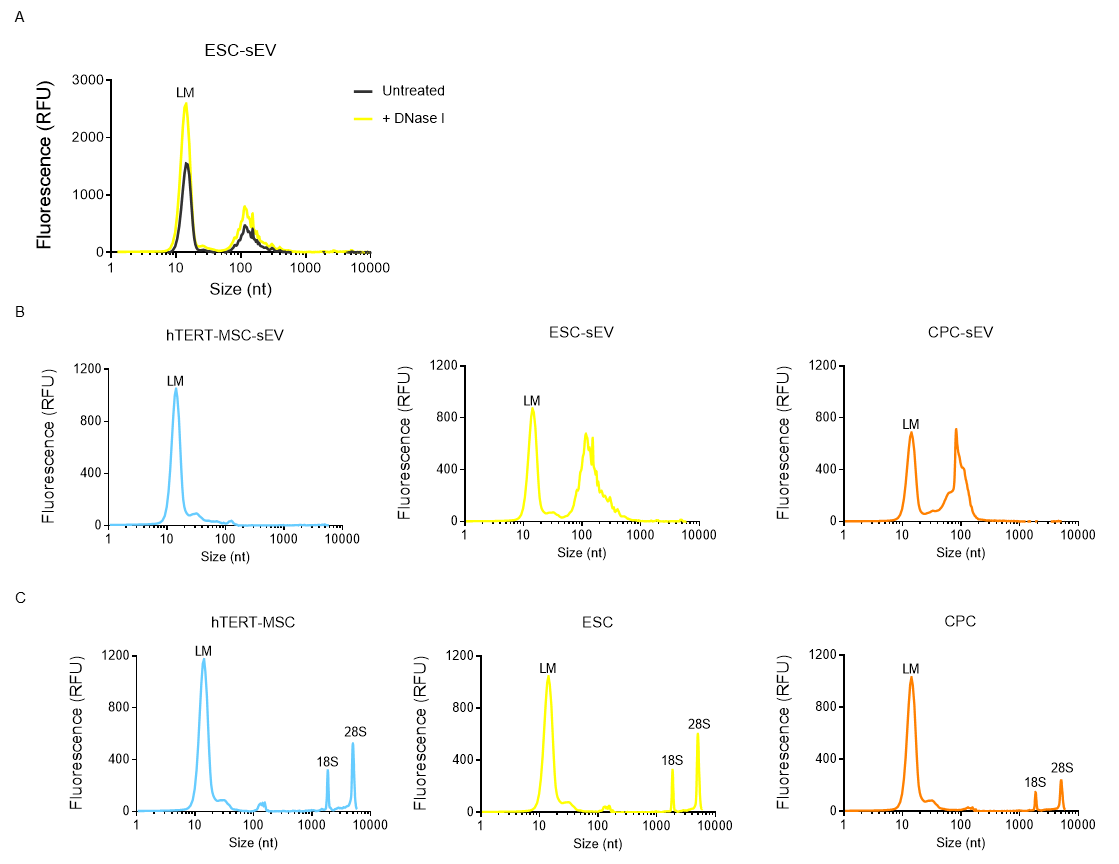


**Supplementary Figure 6. RNA profile in secreted sEV from hTERT-MSC, ESC and CPC and their parental cells. A.** Capillary electrophoresis electropherogram of RNA isolated from ESC-sEV with or without DNase I on-column digestion during the RNA extraction process. No changes in the RNA profile are observed, confirming the absence of contaminating DNA in the samples prepared for RNA sequencing. **B.** Capillary electrophoresis electropherogram of RNA isolated from sEV secreted by hTERT-MSC, ESC and CPC, as indicated, showing mainly detectable small-sized RNA. **C.** Capillary electrophoresis electropherogram of RNA isolated from cell pellets of hTERT-MSC, ESC and CPC, as indicated. The 28S and 18S rRNA peaks are indicated. The same amount of RNA was loaded for both sEV and cells RNA samples. LM, marker used in the capillary electrophoresis run.


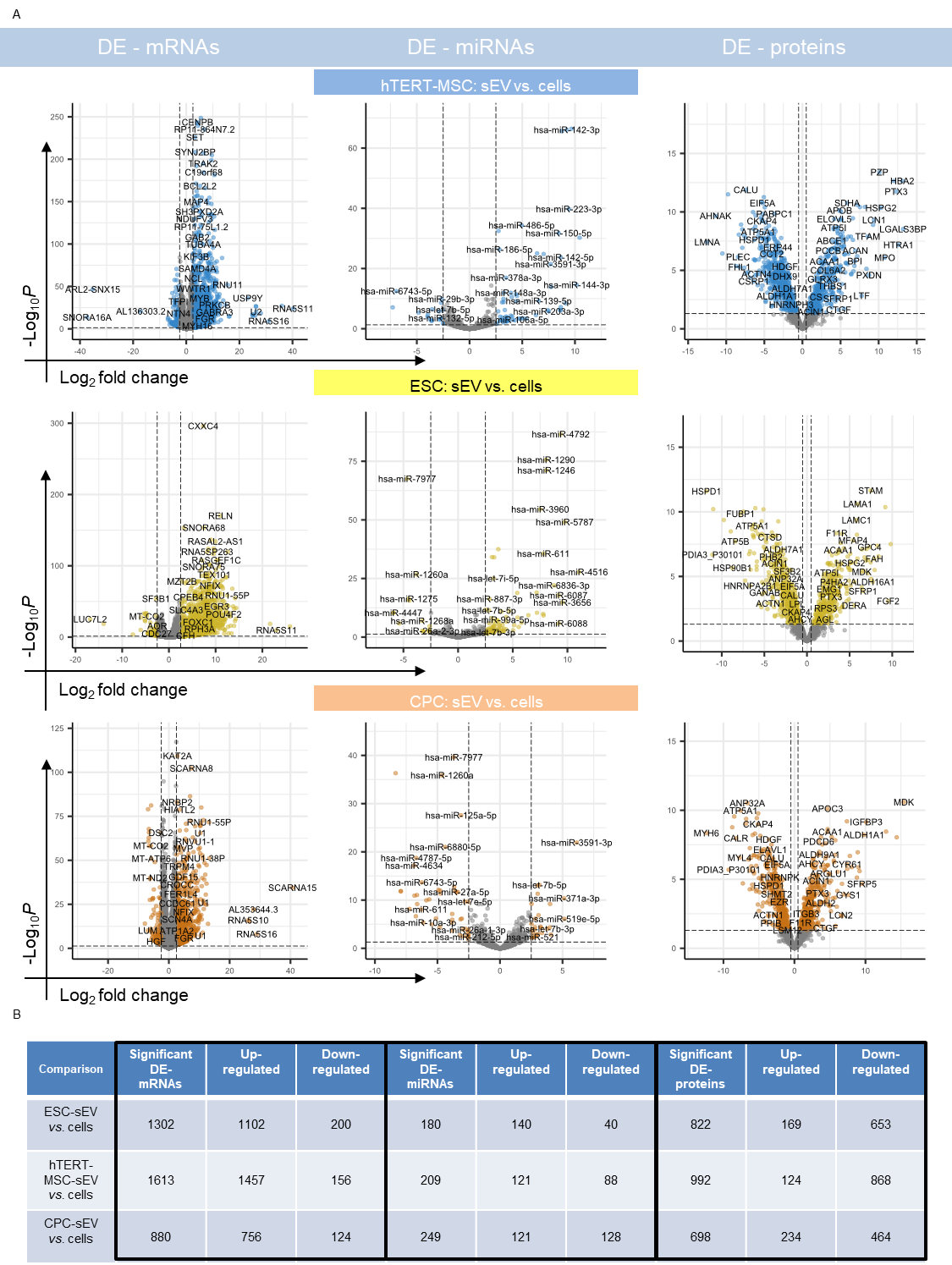


**Supplementary Figure 7. Volcano plots showing differentially expressed mRNAs, miRNAs, and proteins in sEV *vs.* their parental cells. A:** Volcano plots of differentially expressed mRNAs, miRNAs and proteins of sEV *vs.* their respective parental cells. The tabulation for the mRNAs or miRNAs is a p-value < 0.05 and |log2FC| > 2.5 and for the differentially expressed proteins is a p-value < 0.05 and |log2FC| > 0.5. **B:** Summary of the number of differentially expressed up-regulated and down-regulated mRNAs, miRNAs, and proteins from sEV *vs.* parental cell. N=5 for RNA samples and N=3 for protein samples.


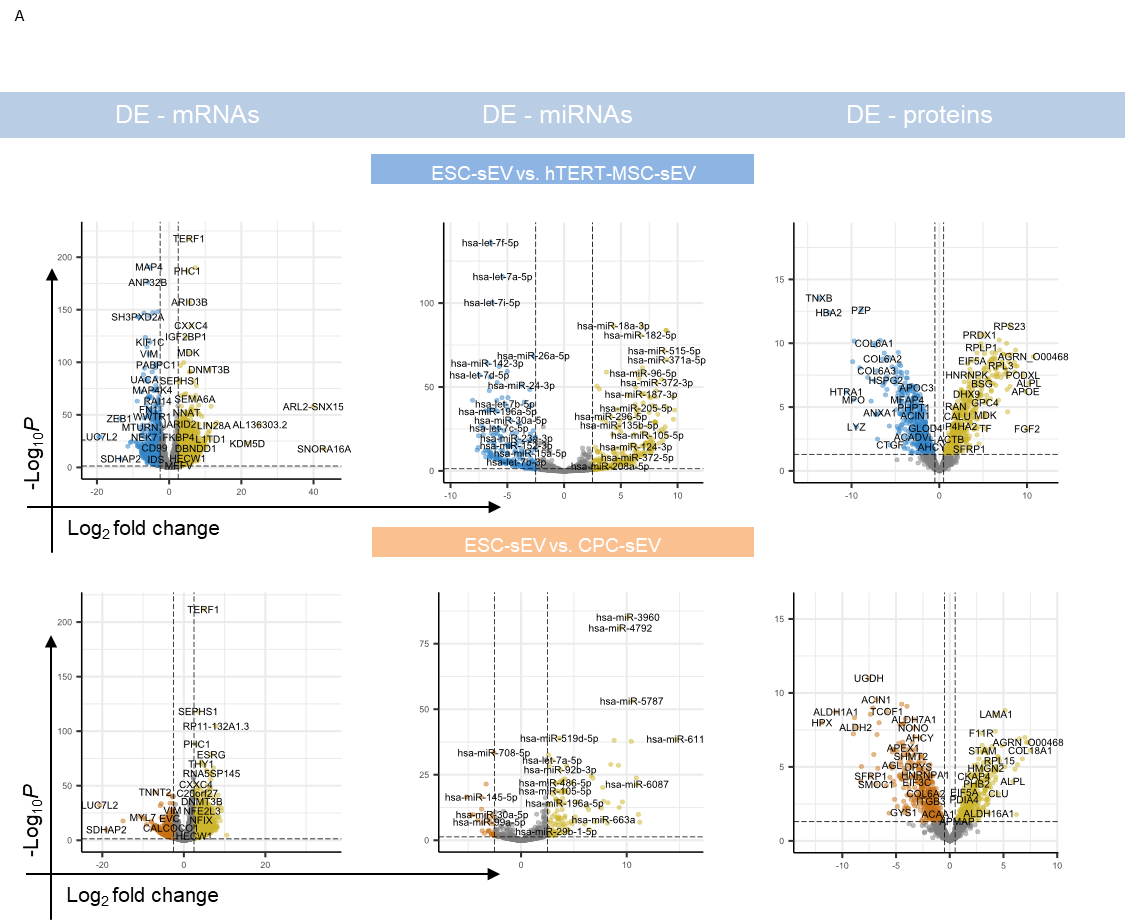


**Supplementary Figure 8. Volcano plots showing differentially expressed mRNAs, miRNAs, and proteins in ESC-sEV *vs.* hTERT-MSC-sEV or CPC-sEV. A:** Volcano plots of differentially expressed mRNAs, miRNAs, and proteins of ESC-sEV *vs.* hTERT-MSC-sEV or CPC-sEV. The tabulation for the mRNAs or miRNAs is a p-value < 0.05 and |log2FC| > 2.5 and for the differentially expressed proteins is a p-value < 0.05 and |log2FC| > 0.5 . N=5 for RNA samples and N=3 for protein samples.


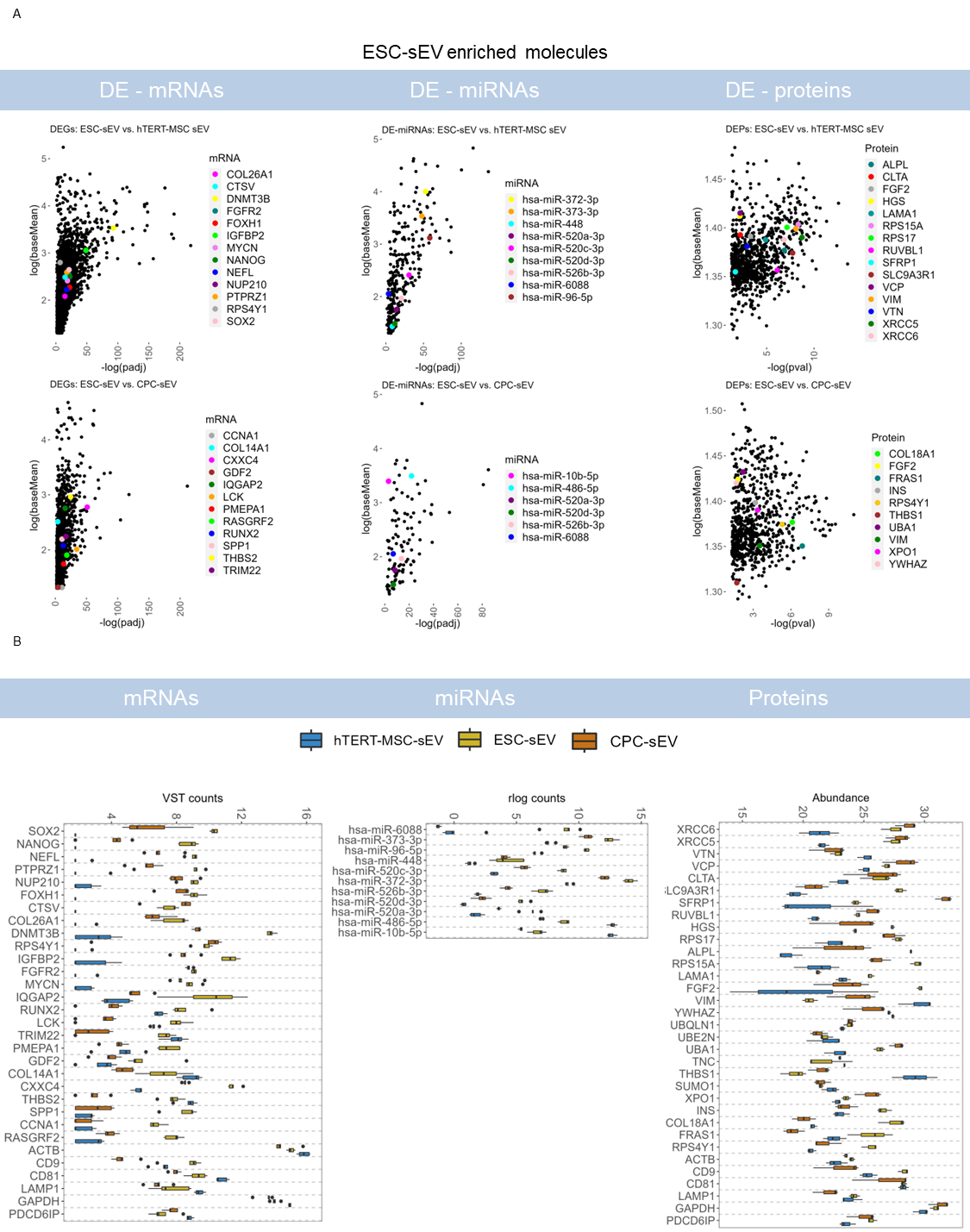


**Supplementary Figure 9. Scatter plots and box plots displaying the expression level of differentially expressed molecules, including mRNAs, miRNAs and proteins, in ESC-sEV compared to hTERT-MSC-sEV and CPC-sEV. A:** Scatter plots showing the abundance and statistical significance of the identified enriched molecules in ESC-sEV compared to hTERT-MSC-sEV (top plots) and CPC-sEV (bottom plots). **B:** Box plots displaying the abundance of a selection of enriched molecules identified in ESC-sEV compared to hTERT-MSC-sEV and CPC-sEV. The housekeeping molecules ACTB, LAMP1, and GAPDH, and the EV markers CD81, CD9 and PDCD6IP (ALIX) are included as references.


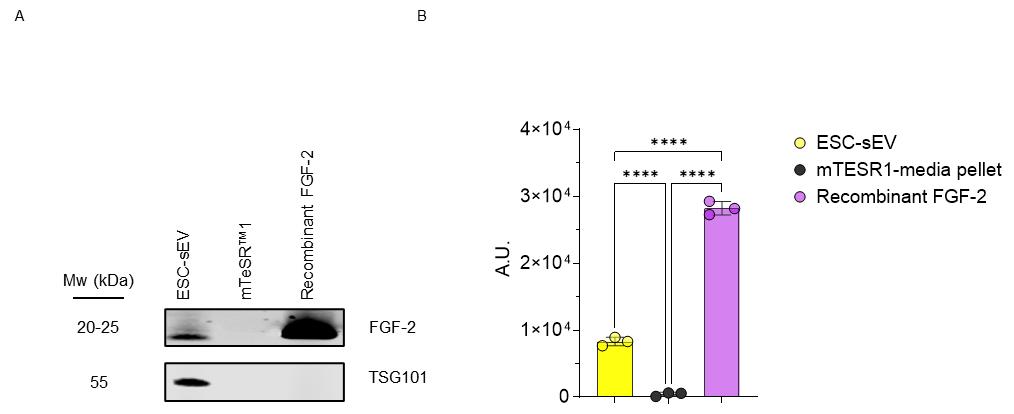


**Supplementary Figure 10. Western blot analysis was employed to investigate the presence of Fibroblast Growth Factor-2 (FGF-2) in ESC-sEV isolated through a serial ultracentrifugation protocol. Additionally, potential protein precipitation from mTeSR™1 culture media following the same ultracentrifugation procedure was examined. A:** Western blot analysis results, depicting the presence of FGF-2 in 0.5 µg of protein extracts from ESC-sEVs (first lane), potentially precipitated proteins from mTeSR™1 culture media after serial ultracentrifugation (second lane), and recombinant FGF-2 (third lane, used as a positive control). TSG101 was used as a sEV loading control. **B:** Densitometric analysis of the Western blot shown in A. Statistical significance was determined using one-way ANOVA, followed by the Tukey post-hoc test (*P<0.05; **P<0.01; ***P<0.001; ****P<0.0001). The dots in the bar graphs represent biological replicates, with N=3. Abbreviations: MW (Molecular weight).

**References**

1. Foo, K.S., et al., *Human ISL1(+) Ventricular Progenitors Self-Assemble into an In Vivo Functional Heart Patch and Preserve Cardiac Function Post Infarction.* Mol Ther, 2018. **26**(7): p. 1644-1659.

2. Thery, C., et al., *Isolation and characterization of exosomes from cell culture supernatants and biological fluids.* Curr Protoc Cell Biol, 2006. **Chapter 3**: p. Unit 3 22.

3. Khan, M., et al., *Embryonic stem cell-derived exosomes promote endogenous repair mechanisms and enhance cardiac function following myocardial infarction.* Circ Res, 2015. **117**(1): p. 52-64.

4. Baker, M., et al., *Use of the mouse aortic ring assay to study angiogenesis.* Nat Protoc, 2011. **7**(1): p. 89-104.

5. Palano, G., et al., *A high-content, in vitro cardiac fibrosis assay for high-throughput, phenotypic identification of compounds with anti-fibrotic activity.* J Mol Cell Cardiol, 2020. **142**: p. 105-117.

6. Li, Q., et al., *Small extracellular vesicles containing miR-486-5p promote angiogenesis after myocardial infarction in mice and nonhuman primates.* Sci Transl Med, 2021. **13**(584).

7. Wu, Q., et al., *Extracellular vesicles from human embryonic stem cell-derived cardiovascular progenitor cells promote cardiac infarct healing through reducing cardiomyocyte death and promoting angiogenesis.* Cell Death Dis, 2020. **11**(5): p. 354.

8. van der Pol, A., et al., *OPLAH ablation leads to accumulation of 5-oxoproline, oxidative stress, fibrosis, and elevated fillings pressures: a murine model for heart failure with a preserved ejection fraction.* Cardiovasc Res, 2018. **114**(14): p. 1871-1882.

9. Grote Beverborg, N., et al., *Phospholamban antisense oligonucleotides improve cardiac function in murine cardiomyopathy.* Nat Commun, 2021. **12**(1): p. 5180.

10. Zacchigna, S., et al., *Towards standardization of echocardiography for the evaluation of left ventricular function in adult rodents: a position paper of the ESC Working Group on Myocardial Function.* Cardiovasc Res, 2021. **117**(1): p. 43-59.

11. Nakayasu, E.S., et al., *Tutorial: best practices and considerations for mass-spectrometry-based protein biomarker discovery and validation.* Nat Protoc, 2021. **16**(8): p. 3737-3760.

12. Savitski, M.M., et al., *A Scalable Approach for Protein False Discovery Rate Estimation in Large Proteomic Data Sets.* Mol Cell Proteomics, 2015. **14**(9): p. 2394-404.

13. Zhang, X., et al., *Proteome-wide identification of ubiquitin interactions using UbIA-MS.* Nat Protoc, 2018. **13**(3): p. 530-550.

14. Love, M.I., W. Huber, and S. Anders, *Moderated estimation of fold change and dispersion for RNA-seq data with DESeq2.* Genome Biol, 2014. **15**(12): p. 550.

15. Kramer, A., et al., *Causal analysis approaches in Ingenuity Pathway Analysis.* Bioinformatics, 2014. **30**(4): p. 523-30.

16. Sticht, C., et al., *miRWalk: An online resource for prediction of microRNA binding sites.* PLoS One, 2018. **13**(10): p. e0206239.

17. Subramanian, A., et al., *Gene set enrichment analysis: a knowledge-based approach for interpreting genome-wide expression profiles.* Proc Natl Acad Sci U S A, 2005. **102**(43): p. 15545-50.

18. Benjamini, Y., A.M. Krieger, and D. Yekutieli, *Adaptive linear step-up procedures that control the false discovery rate.* Biometrika, 2006. **93**(3): p. 491-507.
